# O-sialoglycoprotein Endopeptidase (OSGEP) Suppresses Hepatic Ischemia-Reperfusion Injury-Induced Ferroptosis Through Modulating the MEK/ERK Signaling Pathway

**DOI:** 10.1007/s12033-024-01084-y

**Published:** 2024-03-08

**Authors:** Yuanyuan Tao, Wanqing Zhou, Cheng Chen, Qian Zhang, Zhuoyi Liu, Pingping Xia, Zhi Ye, Chunling Li

**Affiliations:** 1 Department of Anesthesiology, Xiangya Hospital of Central South University, Hunan Province, Changsha, 410008 China; 2 National Clinical Research Center for Geriatric Disorders, Central South University, Hunan Province, Changsha, China

**Keywords:** O-sialoglycoprotein endopeptidase (OSGEP), hepatic ischemia–reperfusion injury (HIRI), Ferroptosis, MEK1/2-ERK1/2 signaling pathway

## Abstract

Hepatic ischemia–reperfusion injury (HIRI) was widely accepted as a critical complication of liver resection and transplantation. A growing body of evidence suggested that O-sialoglycoprotein endopeptidase (OSGEP) was involved in cell proliferation and mitochondrial metabolism. However, whether OSGEP could mediate the pathogenesis of HIRI has still remained unclarified. This study investigated whether OSGEP could be protective against HIRI and elucidated the potential mechanisms. The OSGEP expression level was detected in cases undergoing ischemia-related hepatectomy and a stable oxygen–glucose deprivation/reoxygenation (OGD/R) condition in hepG2 cells. Additionally, it was attempted to establish a mouse model of HIRI, thus, the function and mechanism of OSGEP could be analyzed. At one day after hepatectomy, the negative association of OSGEP expression level with the elevated serum levels of aspartate aminotransferase (AST) and alanine aminotransferase (ALT) was noted. Moreover, it was attempted to carry out gain- and loss-of-function analyses of OSGEP in hepG2 cells to reveal its influences on OGD/R-induced injury and relevant signaling pathways. The findings suggested that OSGEP overexpression significantly protected hepG2 cells against ferroptotic cell death, while OSGEP consumption had opposite effects. Consistent with in vitro studies, OSGEP deficiency exacerbated liver functions and ferroptotic cell death in a mouse model of HIRI. The results also revealed that OSGEP mediated the progression of HIRI by regulating the MEK/ERK signaling pathway. Rescue experiments indicated that ERK1/2 knockdown or overexpression reversed the effects of OSGEP overexpression or knockdown on hepG2 cells under OGD/R condition. Taken together, the findings demonstrated that OSGEP could contribute to alleviate HIRI by mediating the MEK-ERK signaling pathway, which may serve as a potential prognostic marker and a therapeutic target for HIRI.

**Supplementary Information:**

The online version contains supplementary material available at 10.1007/s12033-024-01084-y.

## Introduction

Ischemic reperfusion injury (IRI) is defined as the functional metabolic disorder and structural destruction caused by reperfusion based on ischemic injury after the blood supply of organs and tissues [1]. Hepatic ischemia–reperfusion injury (HIRI) is a serious and harmful surgical complication, typically appearing during hepatobiliary surgery [2], liver transplantation [3] traumatic shock, and severe infection in clinical practice [4]. To date, although tremendous efforts have been made to clarify the potential underlying mechanisms that contribute to HIRI insult, the cellular and molecular events, regulating cell damage after HIRI, have still remained elusive. Therefore, it is important to identify novel molecular regulators for the development of efficacious preoperative protective strategies for HIRI.

sialoglycoprotein endopeptidase (OSGEP) exists in a variety of organisms, and it has a high degree of homology [5]. It is one of the subunits of the kinase endopeptidase and other proteins of small size (KEOPS) complex [6]. Studies have shown that mutated OSGEP may be associated with epilepsy in children, extensive renal tubular involvement, and mitochondrial dysfunction [7, 8]. Knockdown of OSGEP may also inhibit cell proliferation, impair translation of protein, activate DNA damage response, and induce cell death [9, 10]. However, the important role of OSGEP in the pathogenic development of HIRI has still remained elusive.

Ferroptosis is a form of iron-dependent regulating cell death that was first proposed by Dixon et al. in 2012 [11]. The biochemical characteristics of ferroptosis mainly include iron overload and massive lipid peroxidation, followed by changes in the expression levels or activities of glutathione peroxidase 4 (GPX4), glutathione (GSH), and system Xc^−^, as well as imbalance of oxidation system and antioxidant defense system [11, 12]. Ferrous ions and lipid peroxidation are the main driving factors of ferroptosis. Emerging evidence has demonstrated that ferroptosis was identified as a promising target for the prevention and treatment of several liver diseases [13]. Additionally, excessive iron increases the susceptibility to liver damage [14]. Notably, accumulating evidence indicated that ferroptosis is involved in HIRI [15, 16].

Mitogen-activated protein kinases (MAPKs) are composed of the serine/threonine protein kinase family. They are highly conserved in structure and can transmit extracellular signals into cells and nuclei, indicating their critical physiological functions [17]. MAPKs regulate various physiological processes of cells by modulating phosphorylation or dephosphorylation levels of serine and threonine residues [17, 18]. Extracellular signal-regulated kinase (ERK), one critical component of MAPKs, is involved in cell growth, differentiation, and death [19]. Mitogen extracellular kinase (MER) is a known upstream regulator of ERK [19, 20]. A plethora of studies have demonstrated that ERK1/2 may regulate HIRI [21, 22]. Noteworthy, Li et al. experimentally unraveled that YRDC, serving as a subunit of the KEOPS complex by combining with OSGEP, could promote the progression of hepatocellular carcinoma (HCC) via modulating the MEK/ERK signaling pathway [23]. Therefore, there exists a possibility that OSGEP performs its functions by regulating the MEK/ERK signaling pathway.

Hence, the present study aimed to determine the critical significance of OSGEP in HIRI, and to explore whether the potential mechanisms could be involved in modulating the MEK/ERK signaling pathway.

## Materials and Methods

### Clinical Data

Study approval was attained from the Ethics Committee of the Xiangya Hospital of Central South University (Changsha, Hunan Province, China). Biopsy samples were obtained from 35 patients with benign liver disease who underwent hepatectomy. Once laparotomy was completed, it was attempted to collect pre-hepatectomy hepatic biopsies, and completion of reperfusion enabled our team to collect post-hepatectomy hepatic biopsies. The ischemic interval of 15–30 min was considered. Detecting serum contents of alanine transaminase (ALT) and aspartate aminotransferase (AST) was conducted at 1 day after resection. The informed consent was obtained from all patients.

### Determining Serum Contents of ALT and AST

It was attempted to measure serum contents of ALT and AST by commercial kits provided by Jiancheng Bioengineering Institute (Nanjing, China) through an AU5400 analyzer (Olympus, Tokyo, Japan).

### Detection of OSGEP

Extraction of venous blood (5 ml) was performed to detect OSGEP level by a KL-10794H kit (Kalang Co., Ltd., Beijing, China). In addition, the total RNA in biopsy samples was collected via TRIzol reagent provided by Invitrogen (Carlsbad, CA, USA). Then, a PrimeScript RT Reagent kit (Takara, Shiga, Japan) was utilized for synthesizing cDNA, followed by quantification via a SYBR Premix Ex Taq system (Takara).

### Cell Culture

The National Collection of Authenticated Cell Cultures (Shanghai, China) provided HepG2 cell lines. The stably transfected cells were provided by Gemma Gene Company (Shanghai, China). Cultivation of cells was carried out in a Dulbecco’s modified Eagle’s medium (Thermo Fisher Scientific Inc., Waltham, MA, USA) using 1% antibiotics (100 U/mL penicillin G and 100 mg/mL streptomycin) and 10% (v/v) fetal bovine serum in a humidified incubator (5% CO_2_, 37 ℃). The passage cycle was 3–5 days. Prior to oxygen–glucose deprivation/reoxygenation (OGD/R), HepG2 cells underwent pretreatment with ferroptosis inhibitors, Liproxstatin-1 (Lip, S7699, Selleck, TX, USA) or deferoxamine (DFO, Novartis Pharma Stein AG, Switzerland). These inhibitors were dissolved in DMSO at a concentration of 1 μM and incubated for 24 h [24]. In addition, hepG2 cells were treated with 10 µM Erastin (Monmouth Junction, NJ, USA) dissolved in DMSO to induce ferroptosis at room temperature for 24 h prior to OGD/R [25, 26]. Professor Liqing from the Department of Clinical Pharmacology affiliated with Central South University provided OSGEP stable knockdown and overexpression HepG2 cell lines, along with their respective vehicle control.

### Establishment of an OGD/R Model

Seeding of hepG2 cells, which were in the logarithmic growth phase, was performed in 96-well plates (5000 cells/100 μl). A glucose-free DMEM was utilized to replace with the cell culture medium, and after 24 h of incubation, treatment was carried out in an anaerobic condition (94%N_2_, 5%CO_2_, and 1%O_2_) at 37 ℃ for 12 h. After treatment, it was attempted to gently remove the glucose-free DMEM and replace it with a preheated complete medium, followed by its placement in a thermostatic incubator (37 ℃, 5% CO_2_) for 24 h to mimic the reperfusion process.

### Cell Transfection

Design and chemical synthesize of ERK1/2 siRNAs (si-ERK1/2) and ERK1/2 vector (ERK1/2-Vec) were carried out by Gene Pharma Corporation (Shanghai, China), followed by their transfection into the cells via Lipofectamine RNAi Max (Invitrogen). The sequences of the si-ERK1/2 oligonucleotides were summarized as follows: si-ERK1: 5’-AUCAUAAGCAGAACAAACCAU-3’; and si-ERK2: 5’-UAUAUAUACAUCUUUCAUCUG-3’. Before OGD/R, 48-h incubation of all the siRNA and plasmids was performed using the transfection media.

### Cell Viability and LDH Assay

This process followed the instruction of the Cell Counting Kit-8 (CCK-8, Dojindo, Kumamoto, Japan). Briefly, seeding of cells into 96-well plates was carried out (density, 5000 cells/well). After the treatment, 10 μl CCK-8 solution was added to each well to avoid bubbles in the dark, and blank control (only complete medium and CCK-8 solution) was set up and incubated in dark for 1 h at 37 ℃. Then, a microplate reader was utilized for measuring the absorbance of each well (wavelength, 450 nm; Synergy™, New York, NY, USA), followed by calculation of the cell viability in each group.

A commercial kit was utilized for the assessment of LDH release on the basis of instructions provided by the manufacturer (Jiancheng Bioengineering Institute).

### Western Blotting

It was attempted to lyse the cells by RIPA lysis solution with 1% protease inhibitor and phosphatase inhibitor (Beyotime, Shanghai, China). Then, centrifugation was conducted at 10,000 g/min for 30 min at 4℃, and the supernatant could be retained. The total concentration of protein could be detected by a BCA assay kit (Pierce, Rockford, IL, USA). Besides, 12% SDS-PAGE gel was utilized for separating total proteins, and subsequently, 50 µg of protein was transferred to polyvinylidene difluoride (PVDF) membranes (Bio-Rad Laboratories, Hercules, CA, USA). After that, one-hour blocking of membranes with 5% nonfat milk was carried out. Subsequently, it was attempted to incubate membranes with primary antibodies at 4 ℃ overnight. The primary antibodies against OSGEP (ab229859, Abcam), Tubulin (ab7291, Abcam), MEK1/2 (#ab178876; Abcam, Cambridge, UK), ERK1/2 (#4695; Cell Signaling Technology, Danvers, MA, USA), p-MEK1/2 (#ab278723; Abcam), and p-ERK1/2 (#4370; Cell Signaling Technology) were diluted and utilized on the basis of instructions provided by manufacturers.

### Establishment of the HIRI Model

The in vivo experiments were conducted according to the Guide for the Use and Care of Laboratory Animals released by the National Institutes of Health, and approval was attained from the Institutional Review Board of Central South University. OSGEP-specific knockout mice were purchased from Gempharmatech Co. Ltd. Besides, it was attempted to maintain C57BL/6 male mice in specific pathogen-free conditions. After anesthesia with 1% pentobarbital sodium, the liver was exposed using a median abdominal incision, and arteries and veins of the left and middle lobes of the liver were clipped by a non-invasive vascular clamp. Reperfusion could be performed by relaxing the clamp. The hepatic portal vein and blood supply to the left lobe and mid-hepatic lobe of the HIRI group were occluded for 90 min. After this ischemic period, the clamp was released to initiate reperfusion for 6 h. All animal procedures were examined and approved by the Medicine’s Institutional Animal Care and Use Committee of Central South University (Changsha, China; Approval No. 2020sydw0093).

#### Determination of Ferrous Ion Concentration

Preparation of cell samples: We used 2 × 10*^6^ cells in 100 µl of Iron assay buffer, homogenized on ice using a homogenizer or gentle sonication on ice with a few short pulses. Centrifuge at 16,000 g for 10 min at 4 °C to remove insoluble materials and use the supernatant for the assay. Preparation of tissue: The same mass of tissue was taken. Wash tissue in cold PBS. Homogenize tissue in 4 volumes of Iron Assay Buffer using a homogenizer on ice. Centrifuge at 16,000 g for 10 min to remove insoluble materials. Collect the supernatant and transfer to a clean tube.

The ferrous ion concentration was determined according to the instructions of the Iron Assay kit (#ab83366; Abcam). All reagents were balanced to room temperature before use. The steps were briefly described in the following. The incubation (30 min, 37 ℃) of plate in the dark was performed after gently mixing. Then, addition of a 100 μl probe to each well and incubation (30 min, 37 ℃) were carried out. A microplate reader was utilized for measuring the absorbance (wavelength, 593 nm), and the concentration of Fe^2+^ was determined.

#### GPX4 Activity and GSH Assay

The phosphatidylcholine hydroperoxide (a substrate) was utilized for the purpose of detecting GPX4 activity on the basis of the protocol described previously [27]. Homogenization of ischemic liver samples and the collection of supernatant were performed for analyzing GSH through an A06 kit (Jiancheng Bioengineering Institute).

#### Determination of Malondialdehyde (MDA) Concentration

Preparation of cell samples: The same amount of cells (2*10^7^) were collected from each group. The cells were added with 300 μl of lysis buffer and lysed for 30 min. Then centrifuged the lysis buffer at 10,000 g for 10 min at 4 °C. The supernatant was taken for subsequent determination. Preparation of tissue: First, the tissue was washed with PBS, and the PBS on the surface of the tissue was absorbed with filter paper. The same mass of tissue (10 mg) was taken and 500 μl of lysis buffer was added. After homogenization, it was centrifuged at 10,000 g for 10 min at 4 °C. The supernatant was taken for subsequent determination.

The determination of MDA concentration was carried out according to the instructions of the E2019 kit (Applygen, Shanghai, China), which was described previously [28]. All reagents were balanced to room temperature before use. The steps were briefly described in the following. First, it was essential to prepare samples, standards, and TBA solutions. The corresponding components were added to each tube. The tubes were placed in a water bath at 95 ℃ for 30 min and cooled to room temperature on ice. After centrifugation at 10,000 g/min for 10 min, 200 μl supernatant was added to a black 96-well plate. The excitation and emission wavelengths of 535 and 553 nm were, respectively, utilized for measuring fluorescence intensity via a multifunctional microplate reader. Finally, MDA concentration was calculated according to the formula (*Y* = 0.0004X-0.0007, where Y represents MDA concentration, and X indicates fluorescence intensity of detected sample).

#### 12/15-HETE Assay

Through 12/15-HETE ELISA kits (#ab133034/#ab133035; Abcam), it was attempted to measure 12/15-HETE levels on the basis of instructions provided by the manufacturer.

#### Hematoxylin–Eosin (HE) Staining

The HE staining was performed as previously described [29]. Following routine protocols, HE staining was carried out. Tissues were paraffin-embedded, dewaxed, rehydrated, and stained with HE. Slices were observed by an optical microscope, and images were acquired for analysis.

#### Statistical Analysis

At least three repeated and independent experiments were performed to achieve the experimental data that were denoted as mean ± standard deviation (SD). GraphPad Prism 8.0 software was used to carry out statistical analysis. An unpaired t-test was employed for making comparison between two groups. Multiple groups of experimental data were compared using one-way analysis of variance (ANOVA), and the Tukey’s post hoc test of variance was used. Assessment of the correlation between two indicators was performed using Pearson correlation analysis. It was attempted to set statistical significance to *P* < 0.05.

## Results

### OSGEP Expression Level Was Negatively Correlated with HIRI

**Fig. 1:**
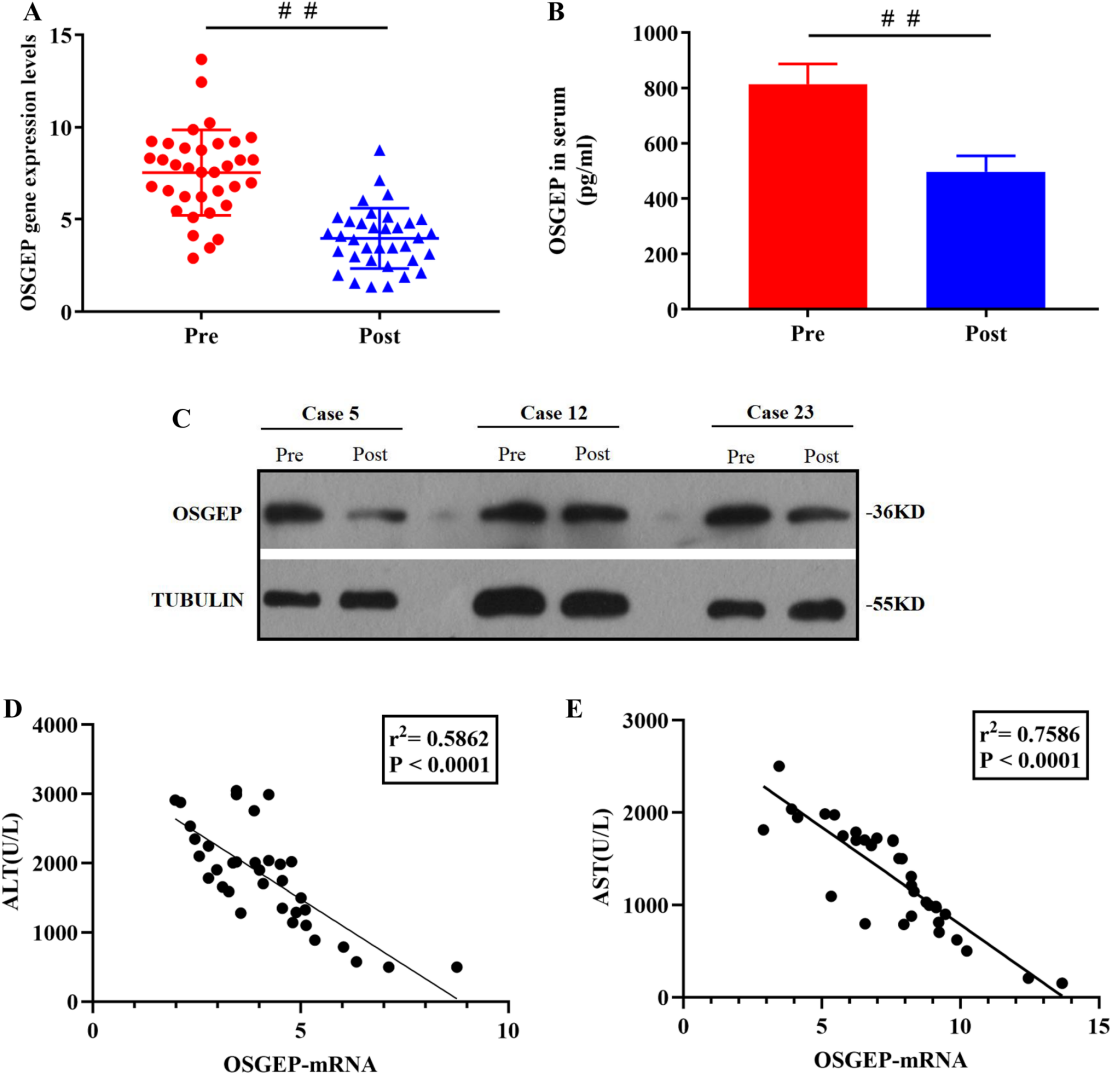
Ischemic reperfusion injury downregulated OSGEP expression level in human ischemic liver. Before hepatic portal occlusion, it was attempted to obtain hepatectomy hepatic biopsies, and prior to abdominal closure, cultivation of post-hepatectomy human biopsies (*N* = 35) was performed at 1.5–2 h post-reperfusion. The ischemic time interval and reperfusion time interval of 15–30 min and 1.5–2 h were considered, respectively. (**A**) Utilization of qRT-PCR for determining OSGEP expression level in human liver; (**B**) ELISA was utilized to determine OSGEP expression level in human serum; (**C**) Western blotting was employed to determine OSGEP protein expression in human liver; (**D**) and (**E**) the native association of ratio of post-hepatectomy OSGEP with serum levels of ALT and AST. Mean ± SD was used to present the data (from three independent experiments). ^##^, *P* < 0.01

To investigate the important role of OSGEP in the pathogenesis of IR-induced liver injury, OSGEP expression level in hepatic biopsies and serum specimens from patients who had undergone ischemia-related hepatectomy was detected. Before hepatic portal occlusion, it was attempted to obtain hepatectomy hepatic biopsies, and prior to abdominal closure, cultivation of post-hepatectomy hepatic biopsies was carried out at 1.5–2 h post-reperfusion. The ischemic interval of 15–30 min was considered. The data suggested that HIRI significantly downregulated OSGEP mRNA level in ischemic hepatic biopsies (Fig. 1A). Consistently, the serum OSGEP level was remarkably reduced post-hepatectomy versus pre-hepatectomy (Fig. 1B). Moreover, the reduction of OSGEP protein expression level was noted in the post-hepatectomy group versus in the pre-hepatectomy control group (Fig. 1C). Notably, the negative association of OSGEP expression level after hepatectomy with serum levels of ALT (Fig. 1D) and AST (Fig. 1E) after hepatectomy was revealed, demonstrating that HIRI was involved in suppressing OSGEP expression level.

### OSGEP Was Involved in the Regulation of OGD/R Injury in hepG2 Cell Lines

Firstly, to establish an in vitro model of HIRI, it was attempted to expose hepG2 cells to OGD treatment for various time points in an anaerobic incubator. To determine the OGD insult to hepG2 cells, a survival rate of approximately 50–60% was considered. The cellular viability was markedly reduced following the prolongation of the OGD, and OGD-12 h was the median lethal time (LT50) and selected for subsequent experiments (Supplementary Fig. S1). After that, it was attempted to determine the optimal reperfusion time, and it was also found that the cellular viability at 24 h after reperfusion was close to 50–60% (Supplementary Fig. S2). Hence, OGD treatment for 12 h and reperfusion for 24 h were selected for subsequent experiments. The similar results were attained by LDH release assay (Supplementary Figs. S3 and S4).

**Fig. 2:**
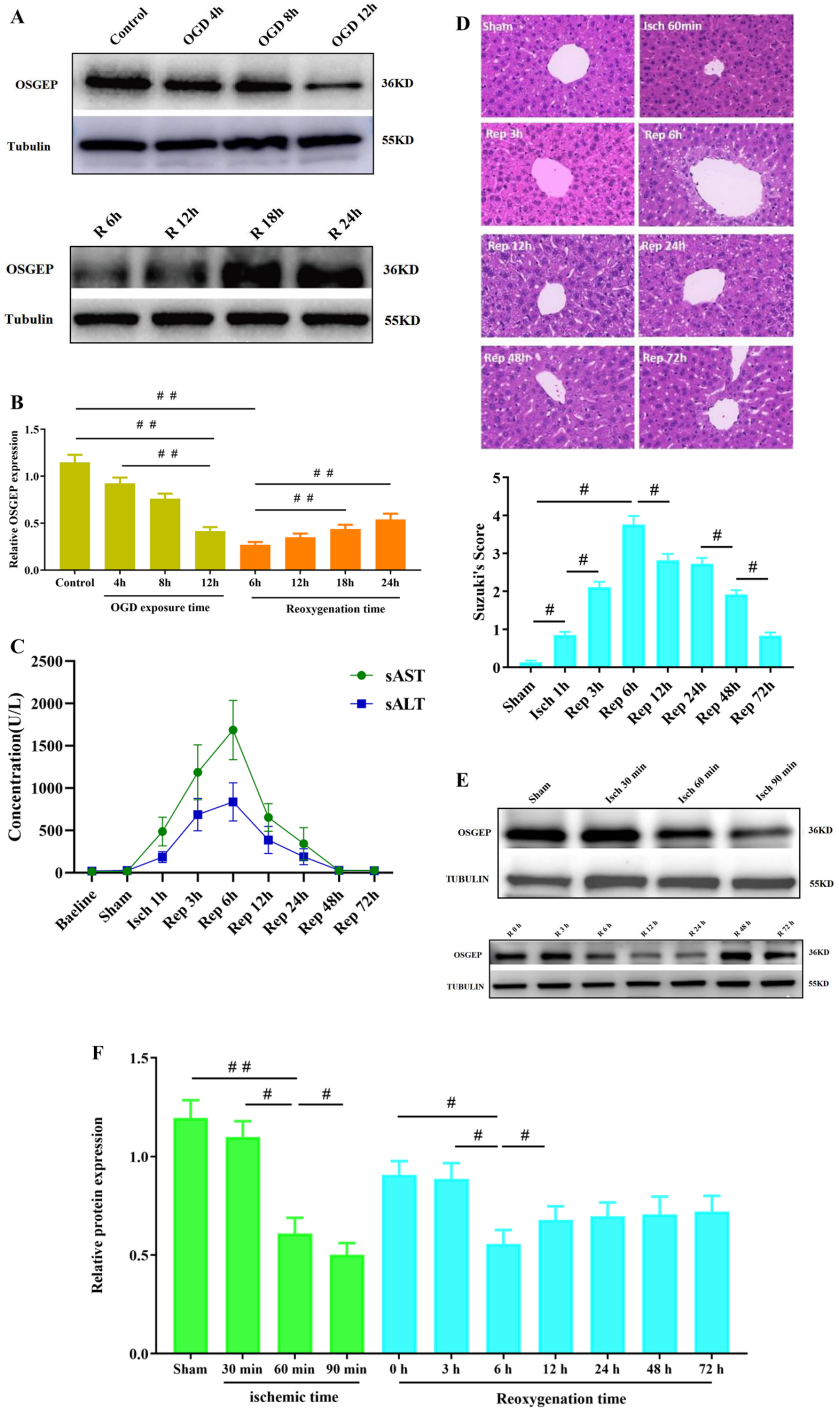
OSGEP was involved in hepG2 cells exposed to OGD/R condition. (**A**) Western blotting of OSGEP protein expression in different OGD exposure time points. (**B**) Western blotting of OSGEP protein expression in different reperfusion time points. (**C**) Determining post-HIRI serum levels of ALT and AST in mice (*n* = 6). (**D**) HE staining was utilized to assess post-HIRI morphological changes in liver tissues. Original magnification, × 200. Scar bar = 100 μm. HE staining was utilized to assess the severity of HIRI via Suzuki’s score. (**E**, **F**) Representative image and Western blotting were employed to indicate OSGEP protein expression in mice at various ischemic and reperfusion time points after HIRI (*n* = 4). Mean ± SD was utilized to present the data (from three independent experiments). ^##^, *P* < 0.01

Secondly, detecting OSGEP expression level in hepG2 cells was carried out under OGD/R condition. It was revealed that OSGEP expression level was gradually downregulated following different OGD treatment time periods and the minimum was present at 12 h after OGD exposure (Fig. 2A). Although the OSGEP expression level was gradually recovered with prolongation of reperfusion time, it was still remarkably lower than the normal level and at least maintained at 24 h after reperfusion (Fig. 2B).

Moreover, a murine HIRI model was established to detect the concentrations of ALT and AST in serum. HE staining was carried out to observe the morphological changes. Compared with the sham group, the concentrations of ALT and AST in the HIRI-treated group were risen and were then reduced. The peak appeared at about 6 h after reperfusion, which subsequently decreased markedly at 72 h after reperfusion (Fig. 2C). Similar trends were noted in the results of histopathology, which were scored by Suzuki criteria in the liver (Fig. 2D). Consistent with in vitro data, OSGEP expression level gradually decreased following different time periods of ischemia and reperfusion, and the minimum appeared at 90 min in ischemic period and 6 h after reperfusion (Fig. 2E, F). Collectively, the results suggested that OSGEP could negatively mediate HIRI.

### OGD/R Injury-Induced Ferroptosis in hepG2 Cell Lines

**Fig. 3:**
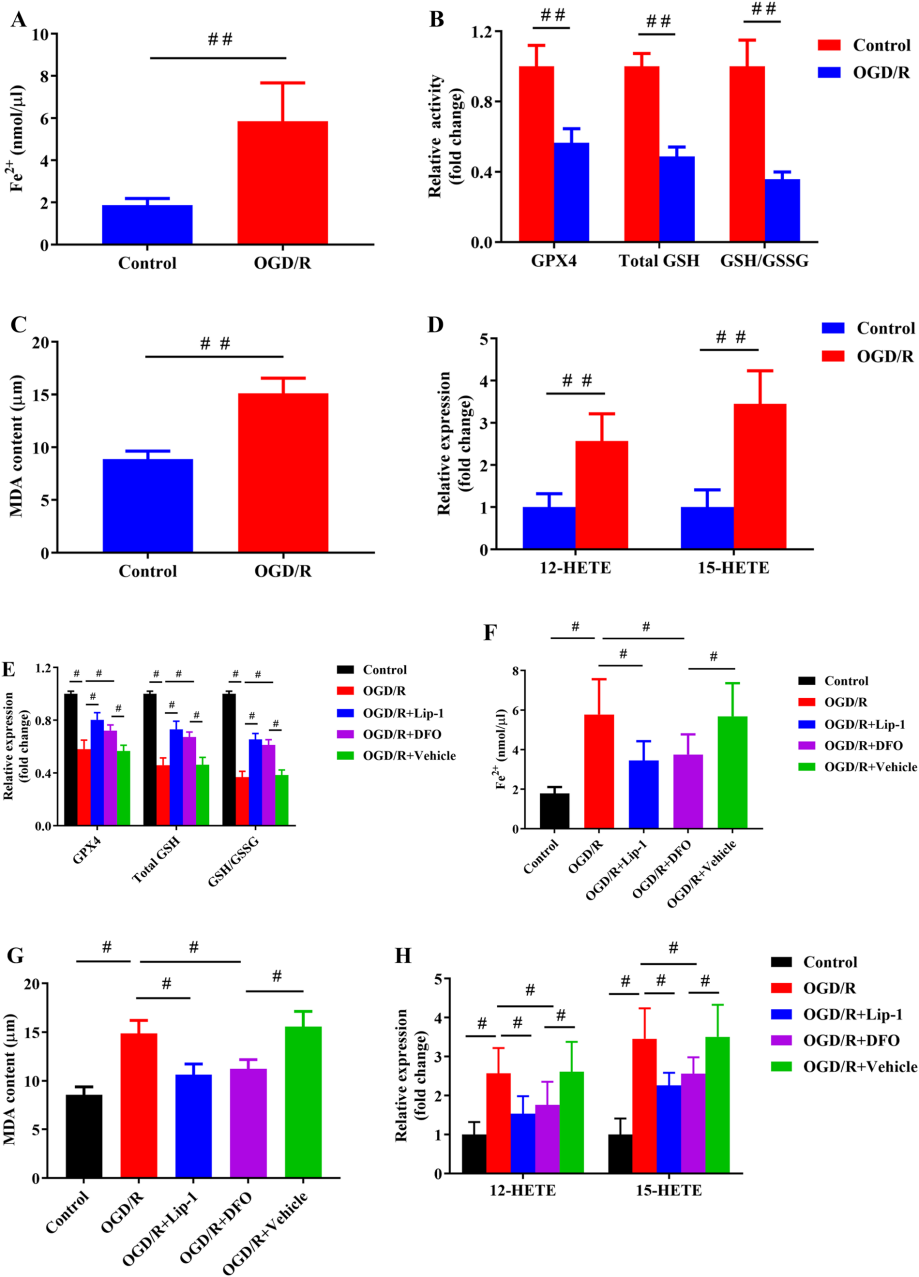
Ferroptosis was present in hepG2 cells affected by OGD/R-induced injury. (**A**) HepG2 cells were subjected to OGD/R injury and were collected to measure iron level. (**B**, **C**) Analysis of GSH/GSSG ratio, GSH level, GPX4 activity, and MDA level by corresponding kits (*n* = 4). (**D**) Utilization of ELISA kits for determining 12-HETE and 15- HETE levels (*n* = 4). Lip and DFO significantly attenuated ischemic reperfusion-induced injury in hepG2 cells. Determination of GSH/GSSG ratio, GSH level, and GPX4 activity (**E**). Determination of iron level (**F**) and cell lipid peroxidation via three assay kits (12-HETE, 15-HETE, and MDA, **G**, **H**). Mean ± SD was used to present the data (from three independent experiments). ^##^, *P* < 0.01; ^#^, *P* < 0.05

Ferroptosis, as a unique regulatory cell death mode, was reported to be driven by iron-dependent lipid peroxidation. To evaluate whether ferroptosis was associated with HIRI, some pivotal and valid ferroptosis-related factors were detected in hepG2 cells with or without OGD/R insult. As depicted in Fig. 3A, iron, a vital factor for ferroptosis, was accumulated in the OGD/R group, rather than in the control group. Additionally, it was found that OGD/R injury noticeably reduced GPX4 activity, GSH level, and GSH/oxidized glutathione (GSSG) ratio (Fig. 3B), while the critical products of lipid oxidation, including the levels of MDA, 15-HETE, and 12-HETE were elevated (Fig. 3C, D).

To further verify the existence of ferroptosis, hepG2 cells were treated with a potent ferroptosis inhibitor, liproxstatin-1 (Lip-1), and a specific iron chelator, deferoxamine (DFO), for 24 h, and they were then subjected to OGD/R. Both Lip-1 and DFO significantly decreased cell death observed in cellular viability and LDH leakage assay (Supplementary Figs. S5 and S6). Moreover, Lip-1 and DFO also partly recovered the GSSG ratio, GSH level, and GPX4 activity (Fig. 3E). Contrarily, Lip-1 and DFO suppressed the iron accumulation, as well as contents of MDA, 12-HETE, and 15-HETE (Fig. 3F–H). Collectively, the role of ferroptosis in HIRI was confirmed.

### OSGEP Could Attenuate HIRI-Induced Ferroptosis

To determine the important role of OSGEP in the progression of HIRI, OSGEP stable knockdown and overexpression hepG2 cell line and its corresponding vehicle control were established by lentiviral vector. In terms of mRNA and protein levels, OSGEP stable knockdown and overexpression cell lines were separately validated versus the vehicle control (Supplementary Figs. S7 and S8). Overexpression of OSGEP in hepG2 cells could promote cellular survival as evidenced by increasing cellular viability and reducing LDH leakage compared with the corresponding vehicle control, while consumption of OSGEP exacerbated cell death (Supplementary Figs. S9 and S10).

**Fig. 4:**
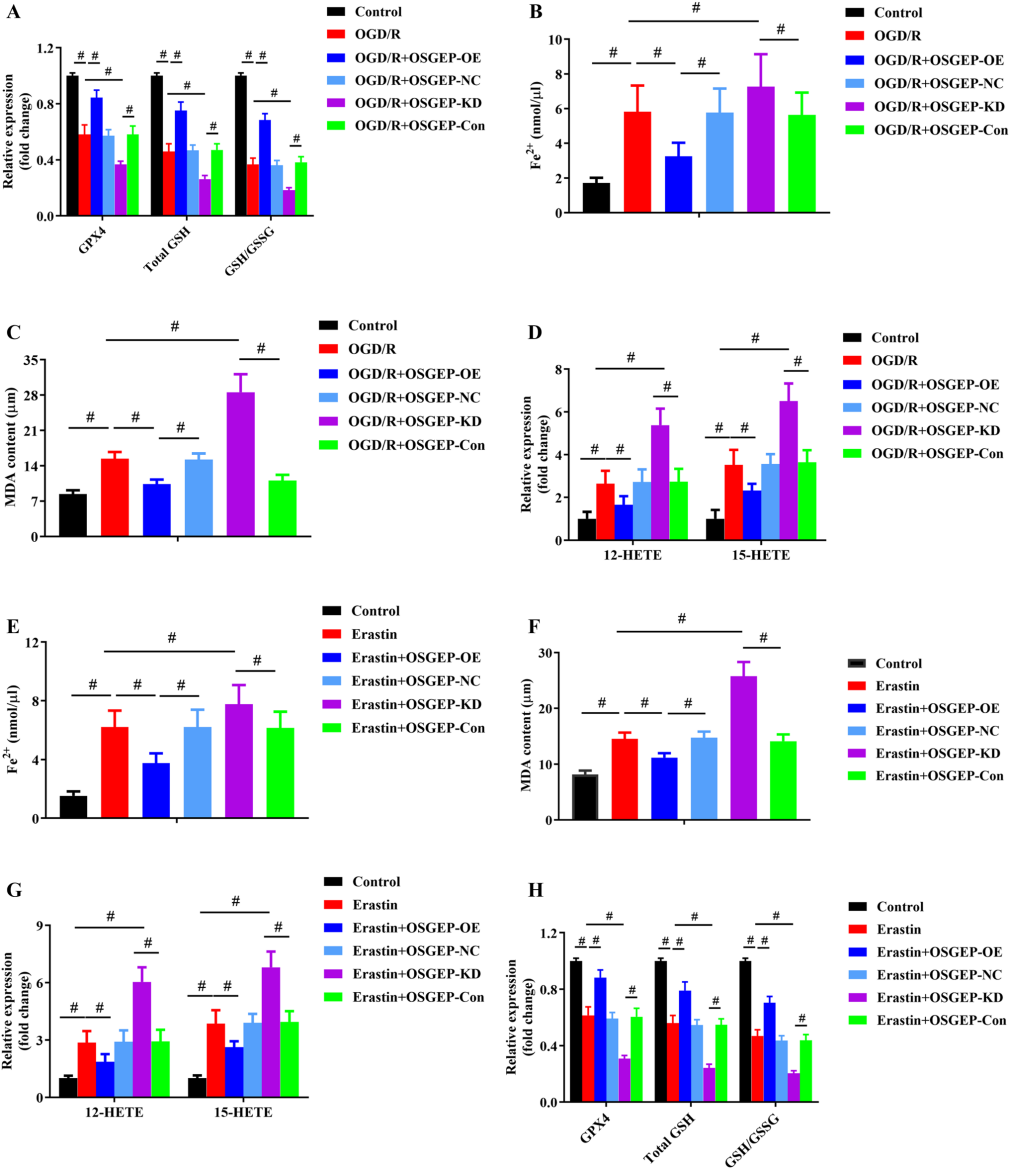
Modulation of OSGEP affected OGD/R or Erastin-induced injury in hepG2 cells. (**A**) Modulation of OSGEP regulated the GSH/GSSG ratio, GSH level, and GPX4 activity in the OGD/R model (*n* = 4). (**B**–**D**) Overexpression of OSGEP alleviated OGD/R-induced iron accumulation, as well as contents of MDA, 12-HETE, and 15-HETE, while OSGEP deletion had opposite effects (*n* = 4). 10 μm Erastin was used to pretreat hepG2 cells to determine the relationship between OSGEP and ferroptosis (*n* = 4). (**E**–**G**) Erastin upregulated Fe^2+^ and MDA concentrations, and increased 12- and 15-HETE levels (*n* = 4). (**H**) The effects of OSGEP overexpression on GSH/GSSG ratio, GSH level, and GPX4 activity with or without Erastin treatment. Mean ± SD was used to present the data (from three independent experiments). ^##^, *P* < 0.01; ^#^, *P* < 0.05

Additionally, overexpression of OSGEP could remarkably elevate GSH/GSSG ratio, GSH level, and GPX4 activity, while it could markedly suppress the levels of iron, MDA, 12-HETE, and 15-HETE. On the contrary, knockdown of OSGEP exhibited opposite effects (Fig. 4A–D).

Erastin is commonly regarded as a potent agonist of ferroptosis, and previous studies have reported that 10 μm Erastin could induce a significant inhibitory effect on the viability of hepG2 cells [25, 26]. The present study revealed that 10 μm Erastin markedly decreased the cell viability and increased LDH leakage in hepG2 cell lines (Supplementary Figs. S11 and S12). Furthermore, the concentrations of iron, MDA, 12-HETE, and 15-HETE were risen (Fig. 4E–G), whereas GPX4 activity and GSH content were reduced in hepG2 cells exposed to Erastin (Fig. 4H). Additionally, susceptibility of hepG2 cells to Erastin was elevated via OSGEP depletion, whereas OSGEP overexpression exhibited the protective effect. Thus, OSGEP could play a negative role in facilitating HIRI-induced ferroptosis.

**Fig. 5:**
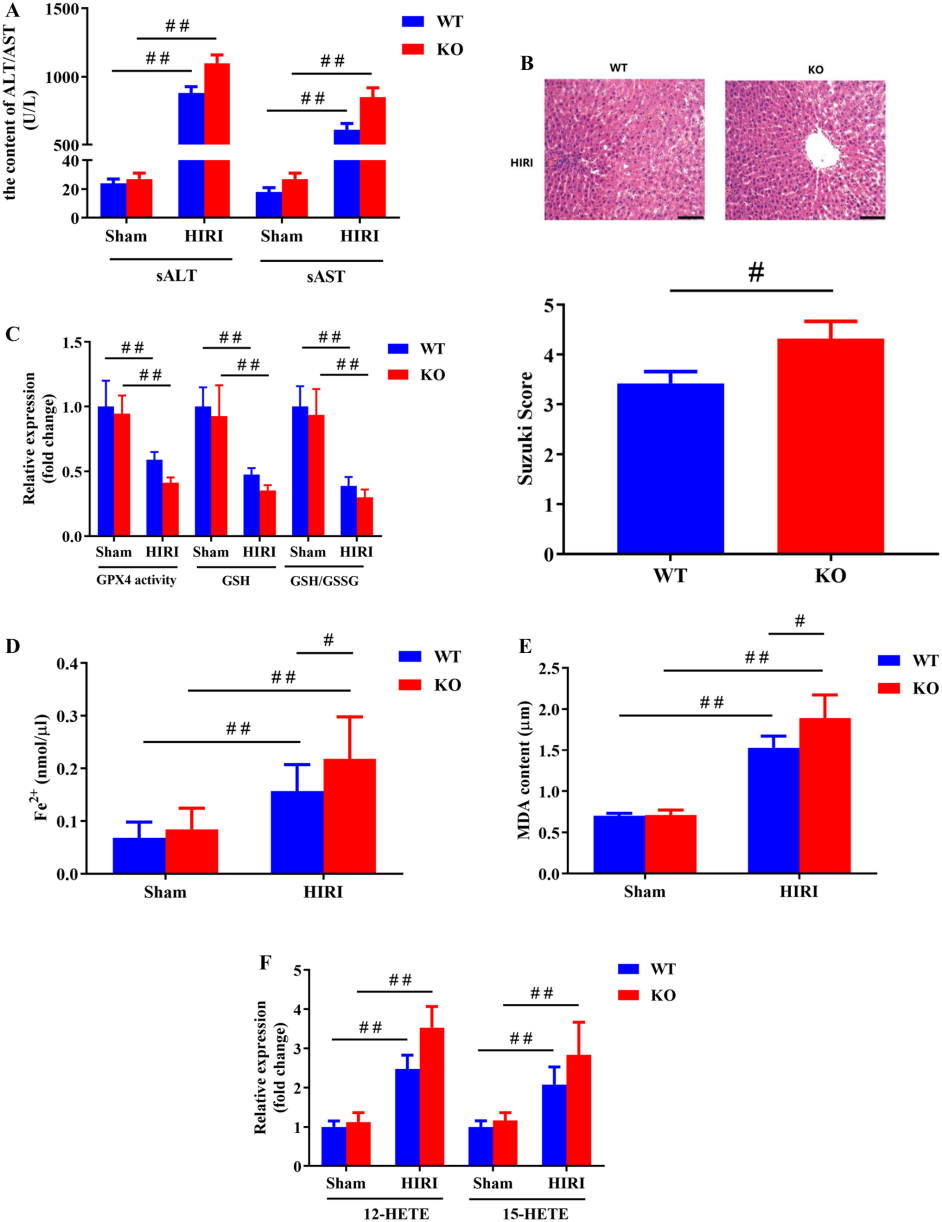
OSGEP deficiency aggravated hepatic ischemic reperfusion injury in mice (*n* = 6). Establishment of a mouse model of HIRI via wild-type and OSGEP-deficient mice. Cultivation of samples at 60 and 360 min after ischemia and reperfusion, respectively. (**A**) Determining serum ALT and AST levels from WT and OSGEP-deficient mice with or without HIRI. (**B**) The ischemic liver tissue was assayed by histological staining (HE). Scale bar was 100 μm. Utilization of Suzuki’s histological score to determine liver damage. (**C**) The GSH/GSSG ratio, GSH level, and GPX4 activity were reduced in OSGEP-deficient mice subjected to HIRI. (**D**–**F**) Concentrations of iron, MDA, 12-HETE, and 15-HETE increased in OSGEP-deficient mice subjected to HIRI. Mean ± SD was used to present the data (from three independent experiments). ^##^, *P* < 0.01; ^#^, *P*  < 0.05

Next, it was attempted to assess the critical function of OSGEP in HIRI via OSGEP-KO mice. In contrast to wild-type (WT) mice, HIRI was dramatically aggravated in OSGEP-deficient mice, as evidenced by the elevated levels of ALT and AST (Fig. 5A) and more severe hepatic architecture, including more sinusoidal congestion, edema, vacuolization or necrosis (Fig. 5B) in OSGEP-KO mice, which were in line with Suzuki’s grading of HIRI. To figure out the influences of OSGEP on cell ferroptosis during HIRI in mice, the analysis of the abovementioned ferroptosis-related factors in ischemic livers was carried out. Importantly, OSGEP deletion significantly reduced GSH/GSSG ratio, GSH level, and GPX4 activity (Fig. 5C), while markedly increased the levels of iron, MDA, 12-HETE, and 15-HETE (Fig. 5D–F) versus WT controls. These results suggested that OSGEP deficiency aggravated HIRI by a mechanism that could be involved in regulating ferroptosis.

### OSGEP Regulated HIRI-Induced Ferroptosis Through the MEK1/2-ERK1/2 Pathway

**Fig. 6:**
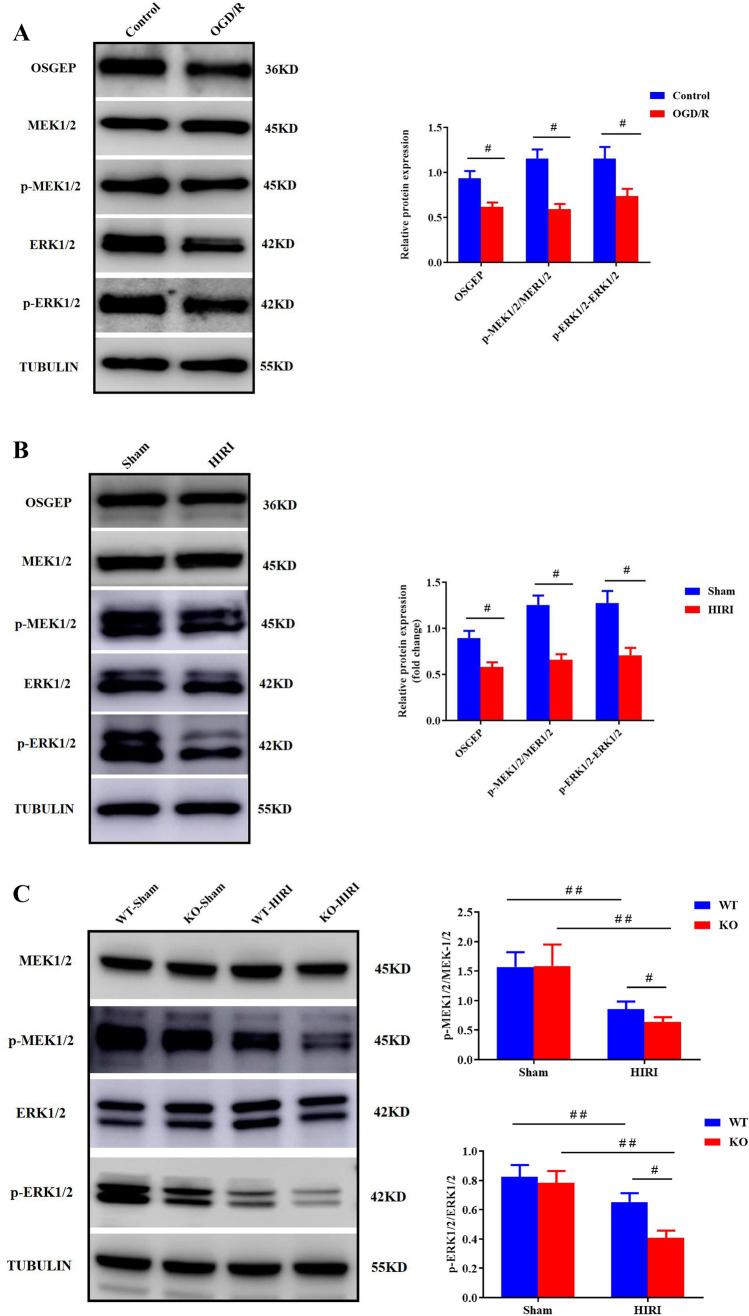

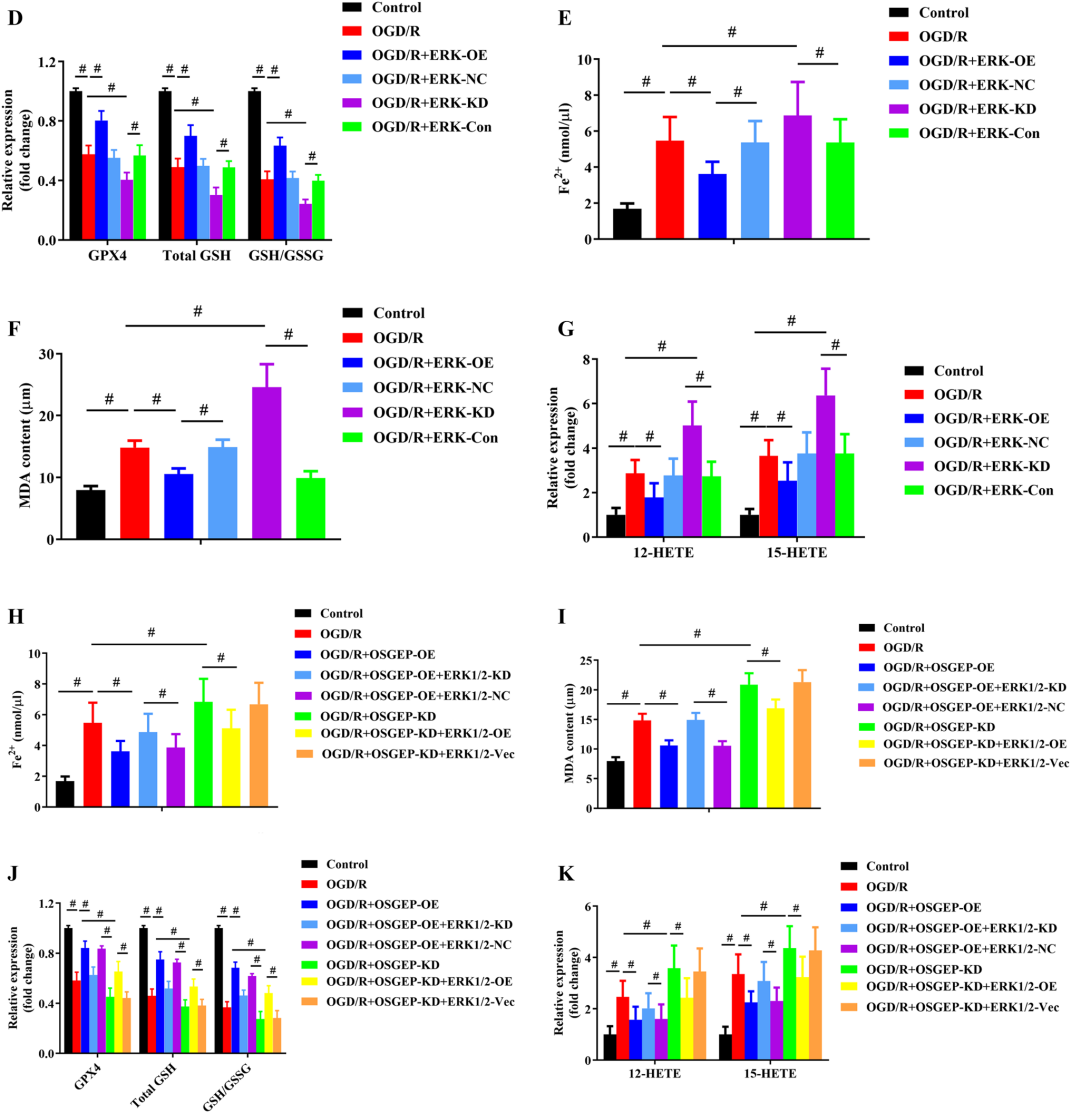
Modulation of OSGEP promoted the MEK/ERK signaling pathway. (**A**) Downregulated levels of OSGEP, p-MEK1/2, and p-ERK1/2 were confirmed by Western blotting in OGD/R-treated hepG2 cells (*n* = 4). (**B**) Downregulated levels of p-ERK1/2, p-MEK1/2, and OSGEP were confirmed by Western blotting in HIRI-treated mice (*n* = 6). (**C**) Levels of p-ERK1/2, OSGEP, and p-MEK1/2 were determined by Western blotting in WT- and OSGEP-deficient mice after HIRI. (**D**) The effects of ERK1/2 overexpression and knockdown on GPX4 activity, GSH level, and the GSH/GSSG ratio in OGD/R-treated hepG2 cells. (**E**–**G**) The effects of ERK1/2 overexpression and knockdown on the levels of iron, MDA, 12-HETE, and 15-HETE were assayed by corresponding kits. (**H**–**J**) The levels of iron, MDA, 12-HETE, and 15-HETE in indicated groups. (**K**) The GSH/GSSG ratio, GSH level, and GPx4 activity were assayed in indicated groups. Mean ± SD was used to present the data (from three independent experiments). ^##^, *P* < 0.01; ^#^, *P* < 0.05

Firstly, the expression levels of the MEK1/2-ERK1/2 pathway in hepG2 cells with or without OGD/R exposure were examined. Compared with the normal control group, the expression levels of total MEK1/2 and ERK1/2 did not significantly vary, while those of p-MEK1/2 and p-ERK1/2 significantly decreased after OGD/R treatment, which was accompanied by OSGEP downregulation (Fig. 6A). Consistently, the expression levels of MEK1/2 and ERK1/2 did not exhibit significant differences between sham and HIRI groups, while the noticeably reduced contents of p-MEK1/2 and p-ERK1/2 in HIRI-treated mice were identified (Fig. 6B). Furthermore, total MEK1/2 and ERK1/2 levels were similar in WT and OSGEP-deficient mice, while the noticeably reduced levels of p-MEK1/2 and p-ERK1/2 were detected in OSGEP-KO mice (Fig. 6C).

Subsequently, pcDNA-ERK1/2 plasmid or ERK1/2 siRNA would be transfected into hepG2 cells to verify the function of ERK1/2 overexpression and inhibition by an in vitro model. The transfection efficiency was confirmed at the protein level by Western blot assay (Supplementary Fig. S13). ERK1/2 overexpression increased GSH/GSSG ratio, GSH level, and GPX4 activity (Fig. 6D), and rescued hypoxia/reoxygenation-induced cell death as determined by the CCK-8 and LDH assays (Supplementary Figs. S14 and S15). Furthermore, ERK1/2 overexpression suppressed lipid peroxidation as indicated by the decreased levels of MDA, 12-HETE, and 15-HETE, as well as the reduced accumulation of ferrous ion. On the contrary, ERK1/2 knockdown had opposite effects (Fig. 6E–G).

To further investigate the relationship between OSGEP and ERK1/2, rescue experiment was performed. The protective effect of OSGEP overexpression on the regulators associated with ferroptotic cell death was abrogated by ERK1/2 knockdown after OGD/R injury. However, ERK1/2 overexpression could rescue the ferroptosis of OSGEP-deleted hepG2 cells from deleterious hypoxia/reoxygenation condition (Supplementary Figs. S16 and S17, Fig. 6H–K). Taken together, OSGEP attenuated HIRI-induced ferroptosis by mediating ERK1/2 pathway.

## Discussion

Notably, HIRI, caused by limited and insufficient blood supply and followed by reperfusion, may exert deleterious effect on hepatic tissue during liver surgery and transplantation, which may seriously influence patients’ prognosis and cause a huge economic burden [1, 2, 4]. At present, there is no broadly accepted therapeutic strategy for mitigating HIRI due to its complex pathophysiological process and interrelated mechanisms. Therefore, it is vital to explore the novel therapeutic targets for minimizing HIRI.

OSGEP, a member of the KEOPS complex, has been regarded as one of the top 10 conserved proteins and its irreplaceable role was confirmed in protein translation, accompanying by high priority for experimental investigation. Pathogenic variants in the *OSGEP* gene could cause a rare autosomal recessive disorder, namely Galloway-Mowat syndrome (GAMOS), which was previously described [27, 28]. Moreover, acute OSGEP knockout in zebrafish larvae had a significantly reduced survival rate and a smaller microcephaly index [10]. Mechanically, OSGEP could interact with YRDC to involve in the modification of N^6^-threonyl-carbamoylation of adenosine 37 (t^6^A) [29]. Moreover, YRDC has been characterized as a new ischemia/reperfusion-inducible protein (IRIP) in renal ischemia/reperfusion operation [30]. Therefore, it was supposed that OSGEP might mediate HIRI. One striking finding of the present study was that OSGEP expression level was correlated with clinical outcomes of patients undergoing hepatectomy. A low OSGEP expression level was associated with worse hepatocellular function in human livers after HIRI. Moreover, OSGEP expression level was downregulated in hepG2 cell lines under OGD/R condition. OSGEP was attributed to protect hepG2 cells against OGD/R and Erastin-induced cell death, whereas knockdown of OSGEP had opposite effects. Hence, these findings suggested that OSGEP could play a critical role in OGD/R-induced hepG2 cell death. However, the potential underlying mechanisms have still remained elusive.

Ferroptosis, a new type of regulated cell death, is induced by combination of iron accumulation, reduction in levels of antioxidants, and plasma membrane damage [11, 12]. It was demonstrated that ferroptosis was involved in HIRI in the liver [31], brain [32], kidney [33], and heart [34]. The present study indicated that ferroptosis inhibition by Lip-1 and DFO remarkably alleviated OGD/R-induced injury in hepG2 cells as evidenced by increasing cellular viability and decreasing LDH release. It is well recognized that the biochemical mechanism of ferroptosis is closely associated with iron overload, the inactivation of GPX4, the reduction of GSH activity, and the accumulation of lipid radicals [35, 36]. Lip-1 and DFO significantly reserved antioxidant system as indicated by promoting GPX4 activity and maintaining GSH/GSSG ratio. Meanwhile, both Lip-1 and DFO significantly decreased the levels of lipid peroxidation products, including 12- and 15-HETE, derived from arachidonic acid (AA)[37], as well as MPO levels. Collectively, the inclusion of ferroptosis in HIRI was confirmed. Parallelly, the results indicated that OSGEP overexpression suppressed the ferroptotic death of hepG2 cells, while there were opposite results for OSGEP depletion in OGD/R condition and Erastin exposure. To further confirm the function of OSGEP, it was attempted to estimate the degree of damage of ischemic livers in OSGEP-deficient mice and wild-type littermates. Strikingly, OSGEP deficiency aggravated serum ALT and AST levels and hepatic architecture, and upregulated some pivotal and valid indicators in ferroptosis regulation. Consistent with clinical data, a negative association between the OSGEP level and the worsening of HIRI was confirmed.

Noteworthily, mitogen-activated protein kinase (MAPK)-extracellular signal-regulated kinase (ERK) pathway, modulating various multicellular functions, has been reported to be associated with HIRI [22, 38]. Besides, in HIRI, the activation of the MEK/ERK signaling pathway prevented the liver from injury by phosphorylation of apoptosis-associated proteins [21]. Although the MEK-ERK pathway has been found to protect hepatic cells against HIRI, few data are available regarding its effect on the ferroptosis in HIRI. In the present study, HIRI was indicated to decrease the phosphorylation of ERK1/2 and its upstream proteins, MEK1/2. Moreover, levels of phosphorylated MEK1/2 and ERK1/2 were elevated by OSGEP overexpression, which is in agreement with attenuating ferroptosis-relative regulators, while OSGEP knockdown had opposite effects. Furthermore, in hepG2 cells treated with ERK1/2 siRNA, the protective effects of OSGEP on cellular viability, LDH release, and ferroptosis-relative regulators were abolished. Contrarily, ERK1/2 reintroduction rescued OSGEP-depleted hepG2 cells from HIRI-induced ferroptotic cell death. These findings suggested that the levels of phosphorylated MEK1/2 and ERK1/2 were risen by OSGEP, and then attenuated HIRI via activating the MEK/ERK signaling pathway. To date, no study has concentrated on OSGEP functioning as a potential protective factor to antagonize HIRI-induced ferroptosis by regulating the MEK/ERK signaling pathway. Additionally, several studies have proven that the MEK/ERK signaling pathway regulated ferroptosis in hemorrhagic stroke [39] and pancreatic cancer [40]. Our previous study had also demonstrated that HCC was progressed through YRDC via activating the MEK/ERK signaling pathway [23]. Hence, the present study revealed that OSGEP could attenuate HIRI via activating the MEK/ERK signaling pathway, thereby inhibiting ferroptotic cell death.

The major limitation of the present study was that hepG2 cell line is a type of human hepatoma cells. Although several studies have cultured it under OGD/R condition to imitate in vitro HIRI, the primary hepatocytes should be used to provide a comprehensive molecular mechanism of OSGEP in HIRI. The correlation between the MEK/ERK signaling pathway and ferroptosis needs to be further investigated.

## Conclusions

In conclusion, the negative regulatory role of OSGEP in progressing HIRI was confirmed, and its overexpression could relieve such injury via activating the MEK/ERK signaling pathway, thereby attenuating ferroptosis in the liver. These findings may assist clinicians to explore the pathogenesis of HIRI. The therapeutic function of OSGEP in alleviating HIRI, particularly in patients undergoing liver resection and transplantation, is noteworthy.

## Supplementary Information

Below is the link to the electronic supplementary material.

Supplementary file1 (DOC 2616 KB)

## Data Availability

The datasets used and/or analyzed during the current study are available from the corresponding author on reasonable request.
