# Supplementary material for: O-sialoglycoprotein Endopeptidase (OSGEP) Suppresses Hepatic Ischemia-Reperfusion Injury-Induced Ferroptosis Through Modulating the MEK/ERK Signaling Pathway
Source: Mol Biotechnol. 2024 Mar 8;67(2):689–704. doi: 10.1007/s12033-024-01084-y (PMC11711258; doi:10.1007/s12033-024-01084-y)
Supplement: Supplementary file 1 — Supplementary file1 (DOC 2616 KB) [file 12033_2024_1084_MOESM1_ESM.doc]

**Supplement figures**

**S1.**

**
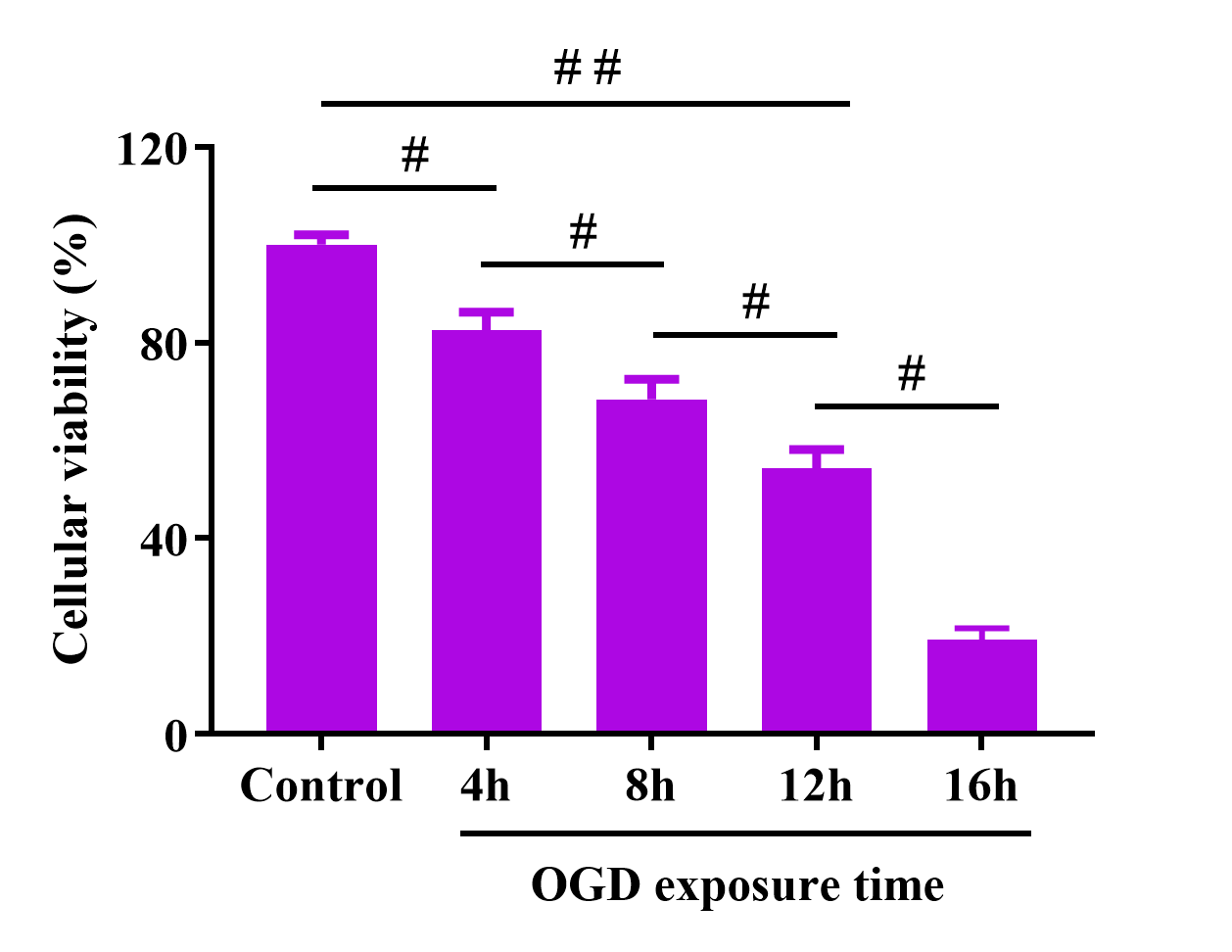
**

**S2.**

**
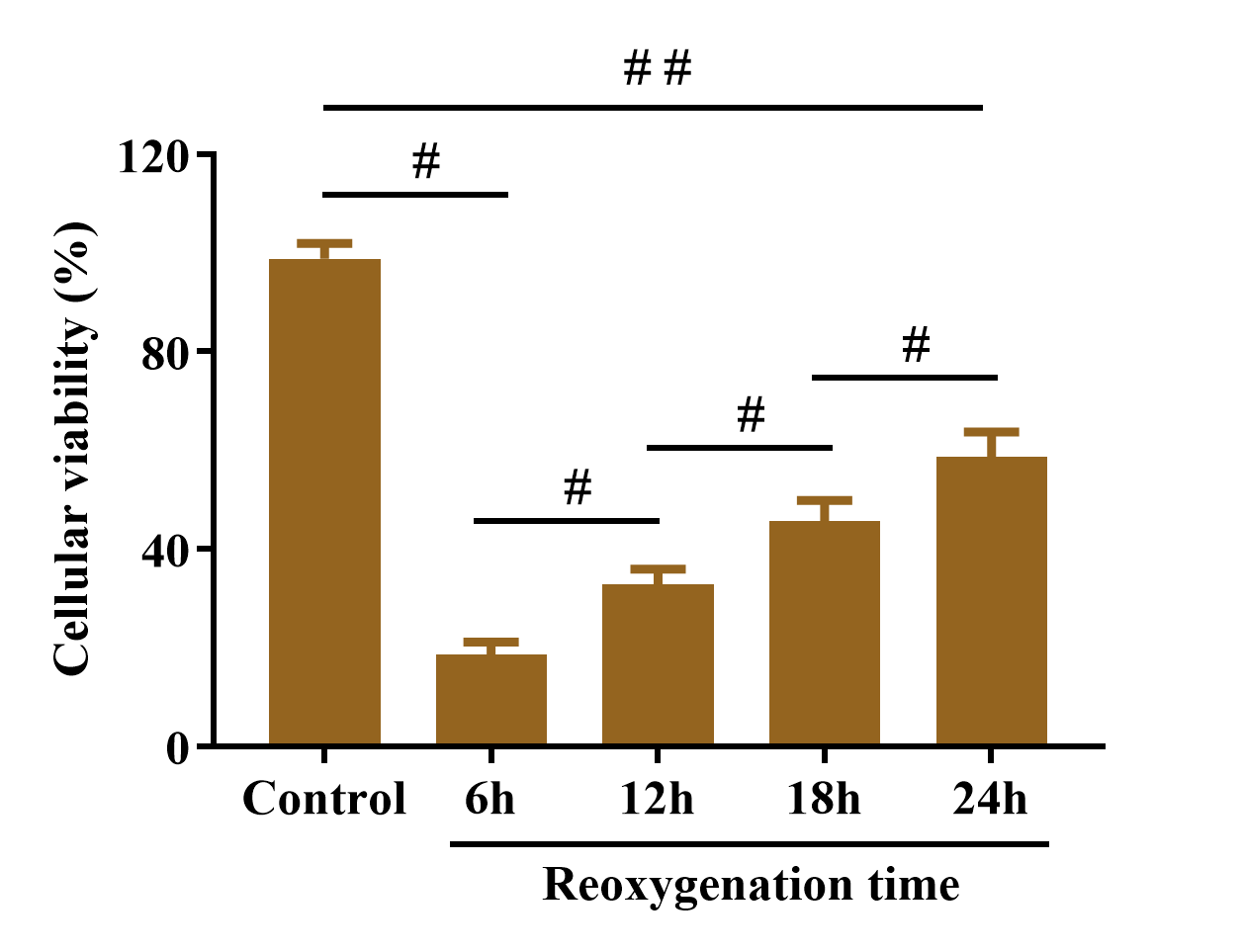
**

**S3.**

**
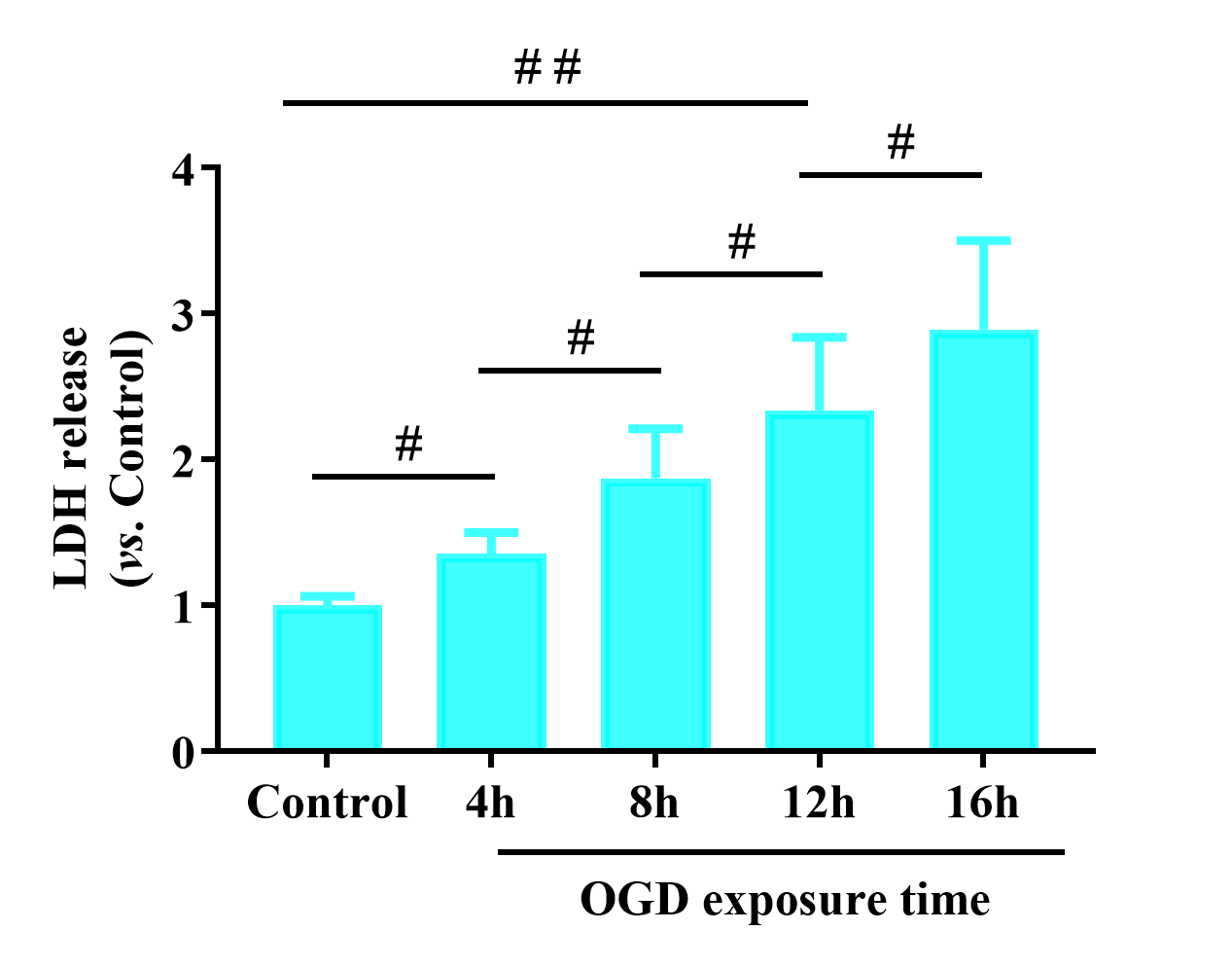
**

**S4.**


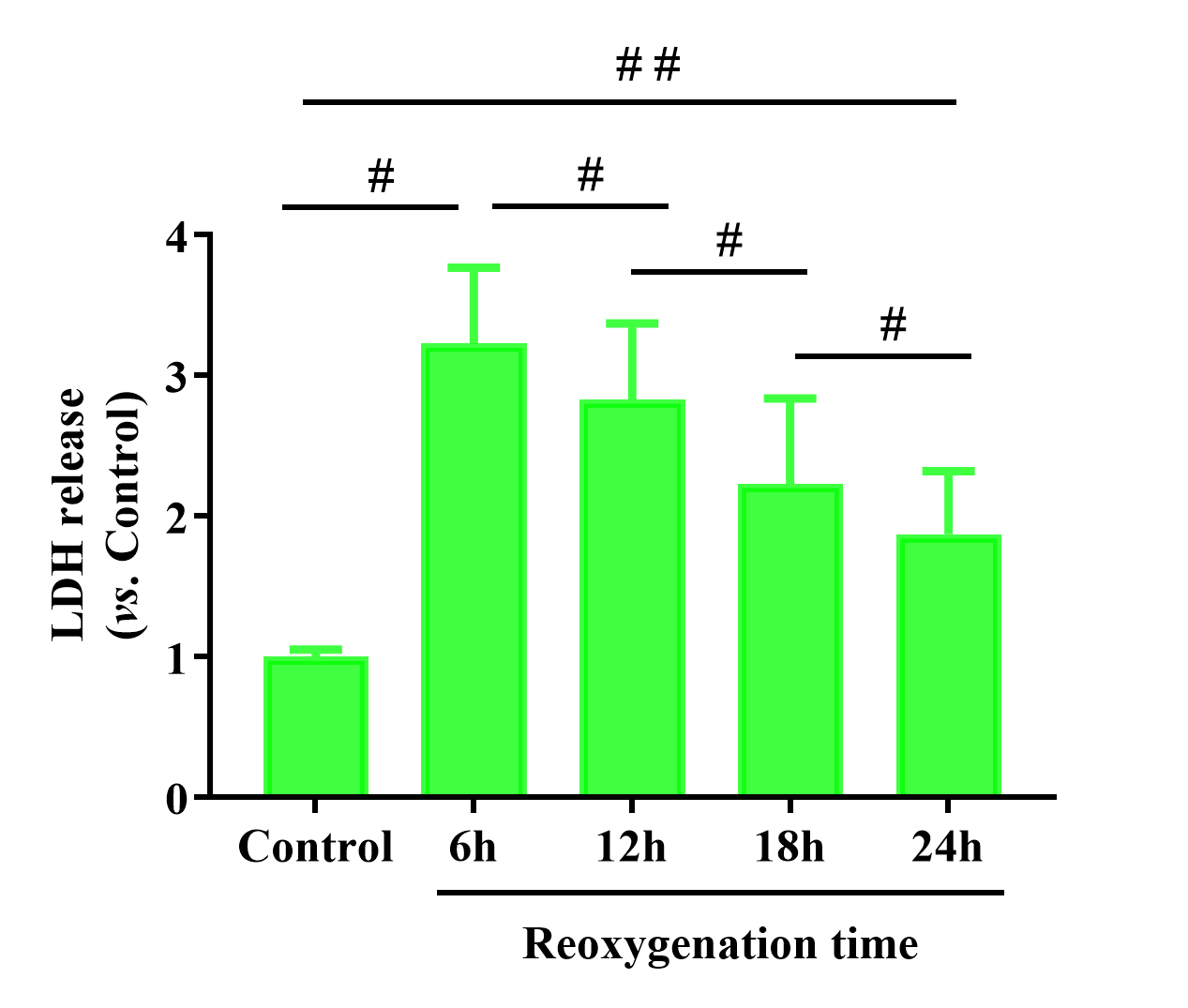


**(S1)** and **(S2)** The optimal OGD and reperfusion time could be selected based on CCK-8 assay. **(S3)** and **(S4)** The similar results were attained by LDH release assay.

**S5.**

**
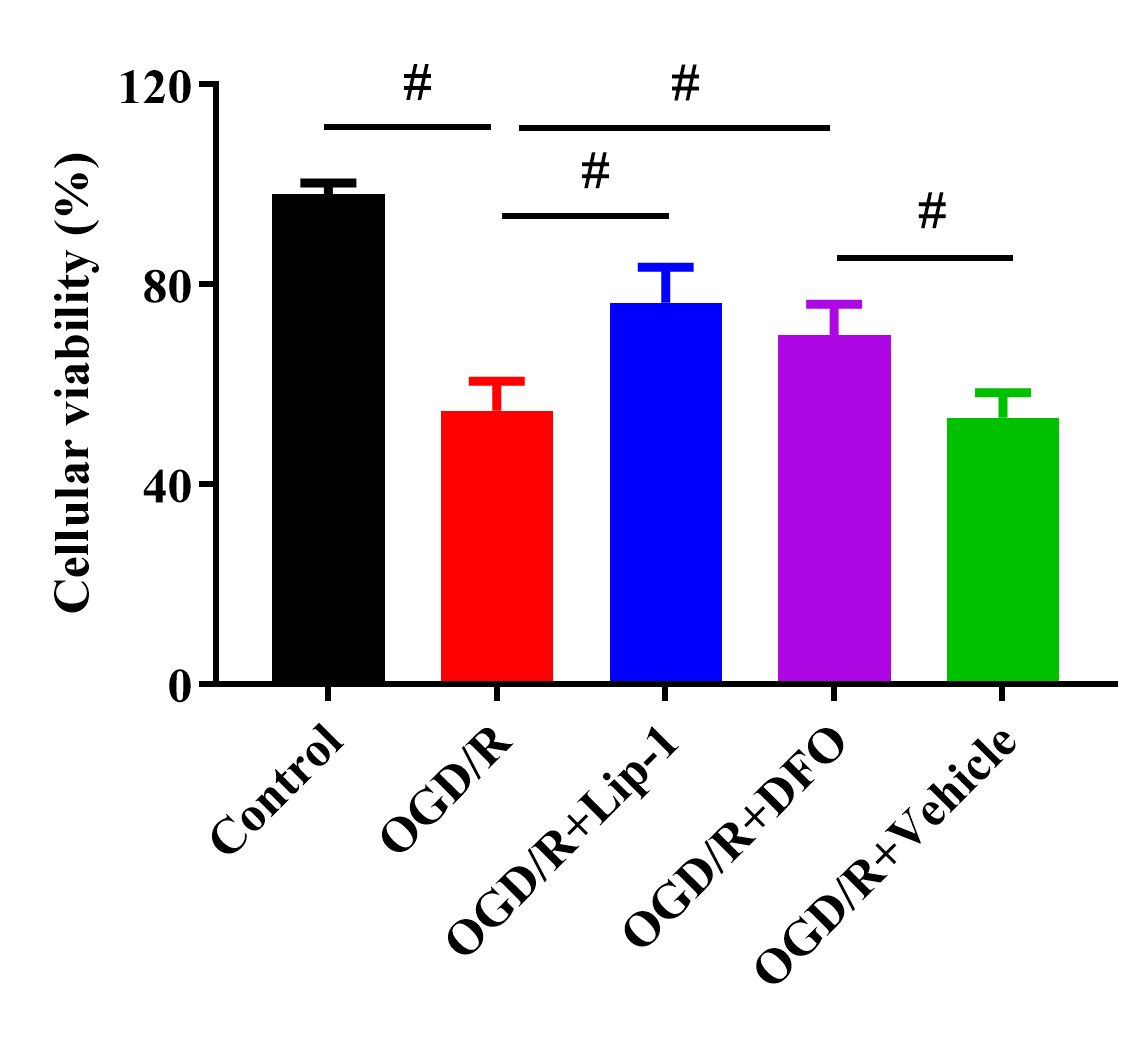
**

**S6.**

**
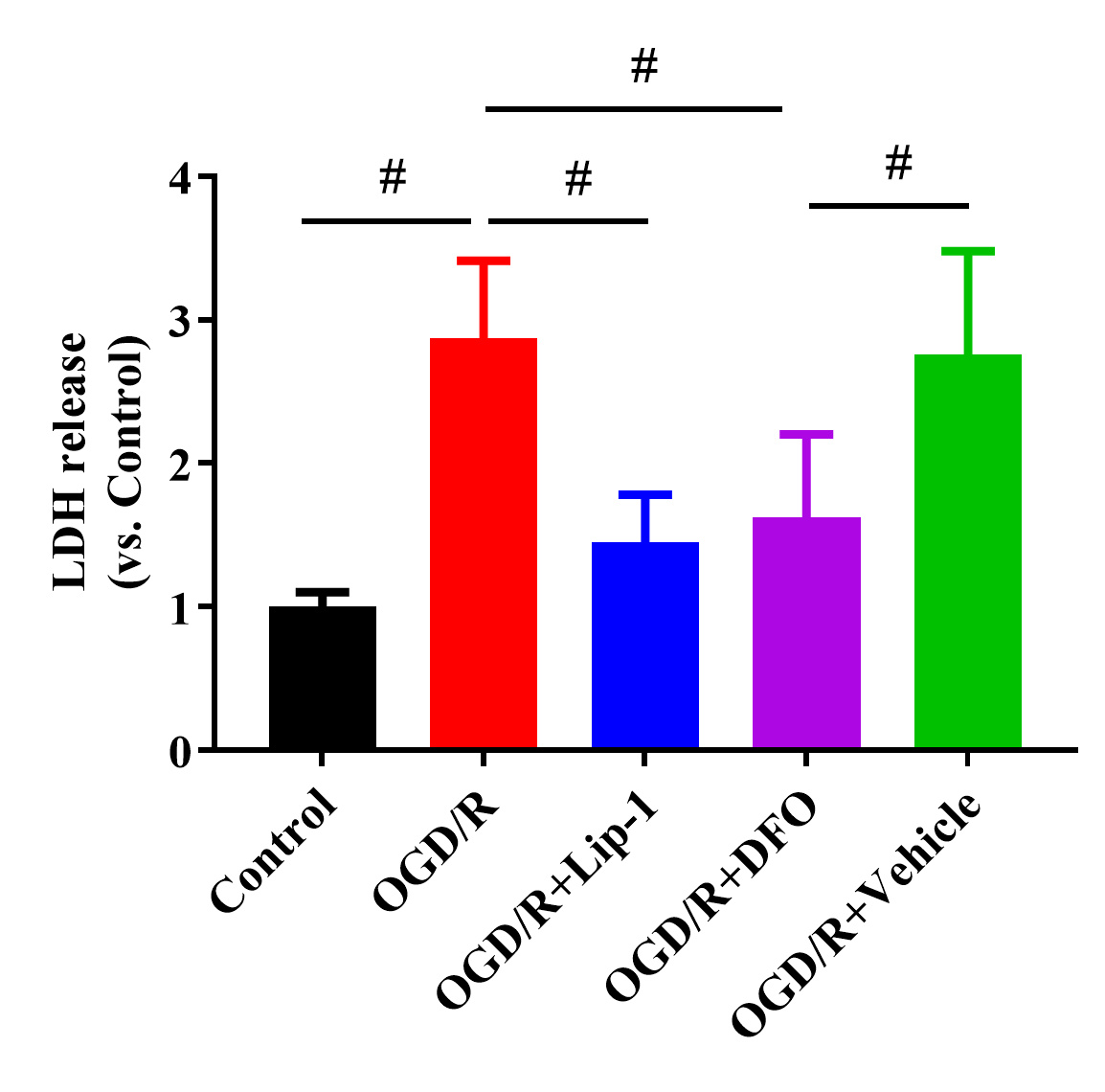
**

**(S5)** and **(S6)** Lip and DFO protected cells against OGD/R exposure as shown by elevating cellular survival and reducing LDH leakage.

**S7.**

**
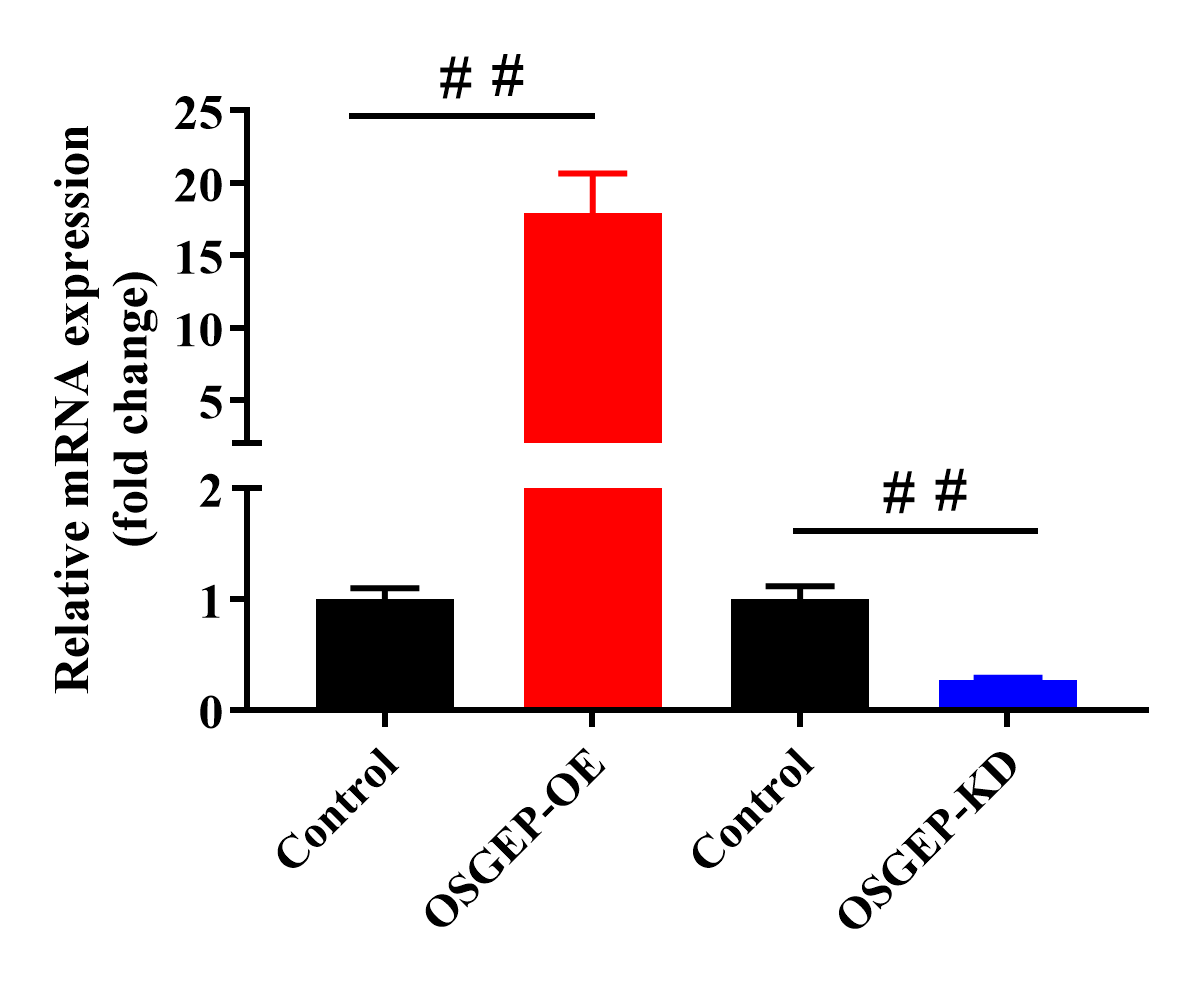
**

**S8.**


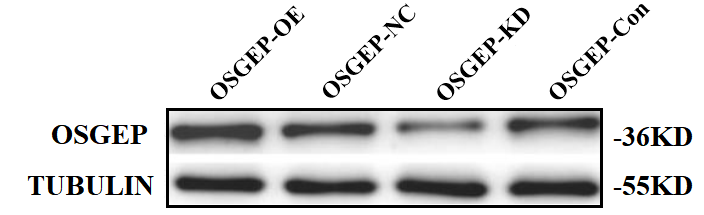


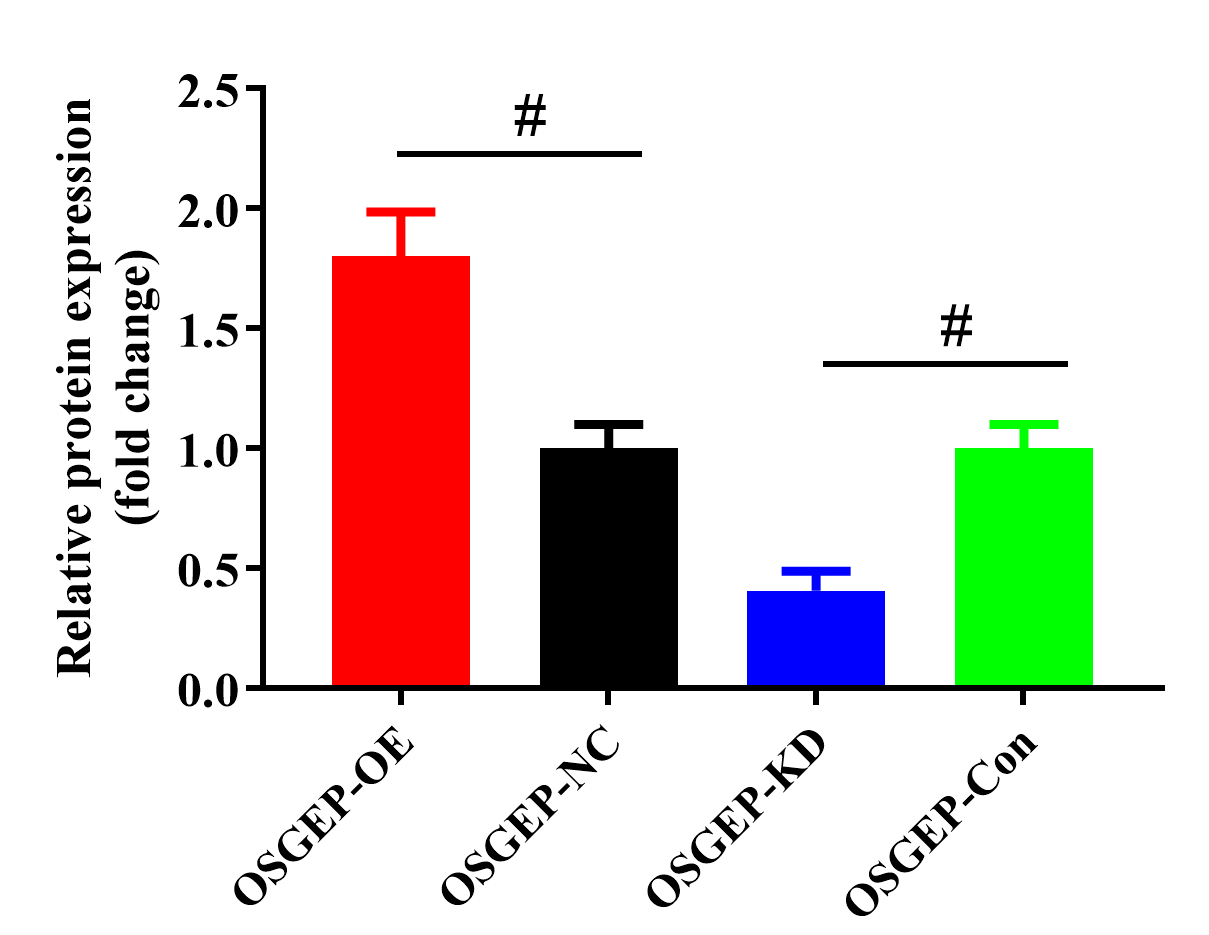


(**S7**) Utilization of RT-PCR for determining the knockdown and overexpression efficiencies of OSGEP. (**S8**) Utilization of Western blotting for determining OSGEP knockdown and overexpression efficiencies.

**S9.**

**
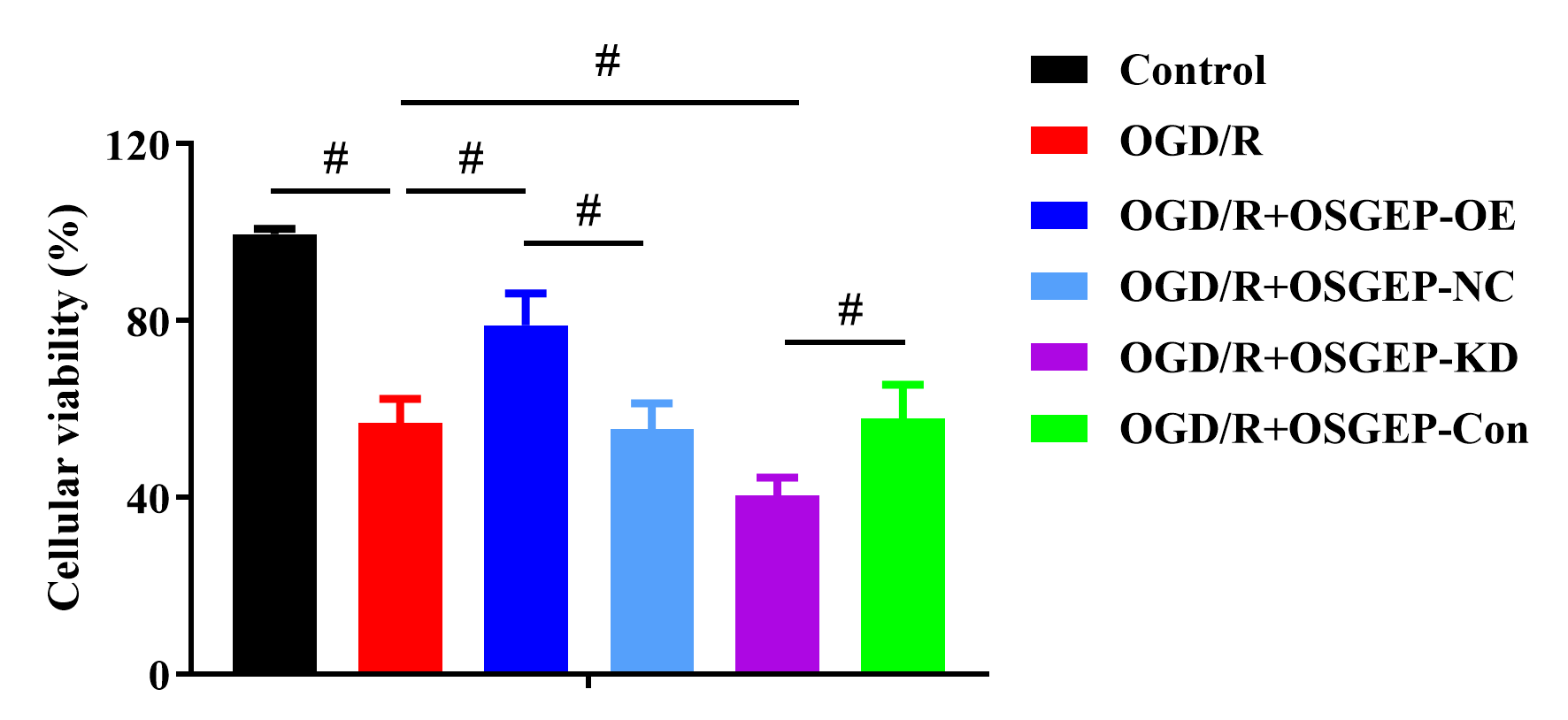
**

**S10.**

**
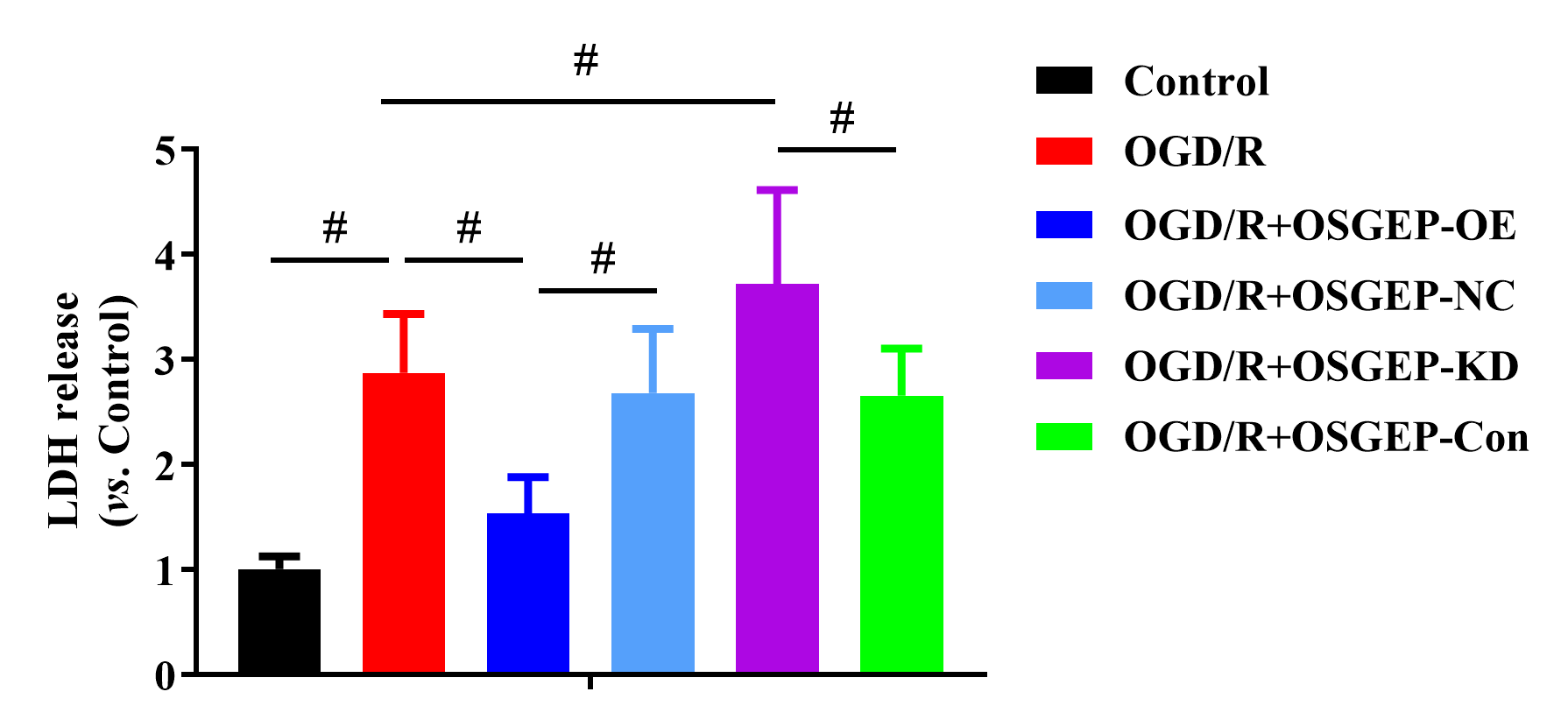
**

(**S9**) and (**S10**) Overexpression and consumption of OSGEP affected the cellular viability and LDH release

**S11.**

**
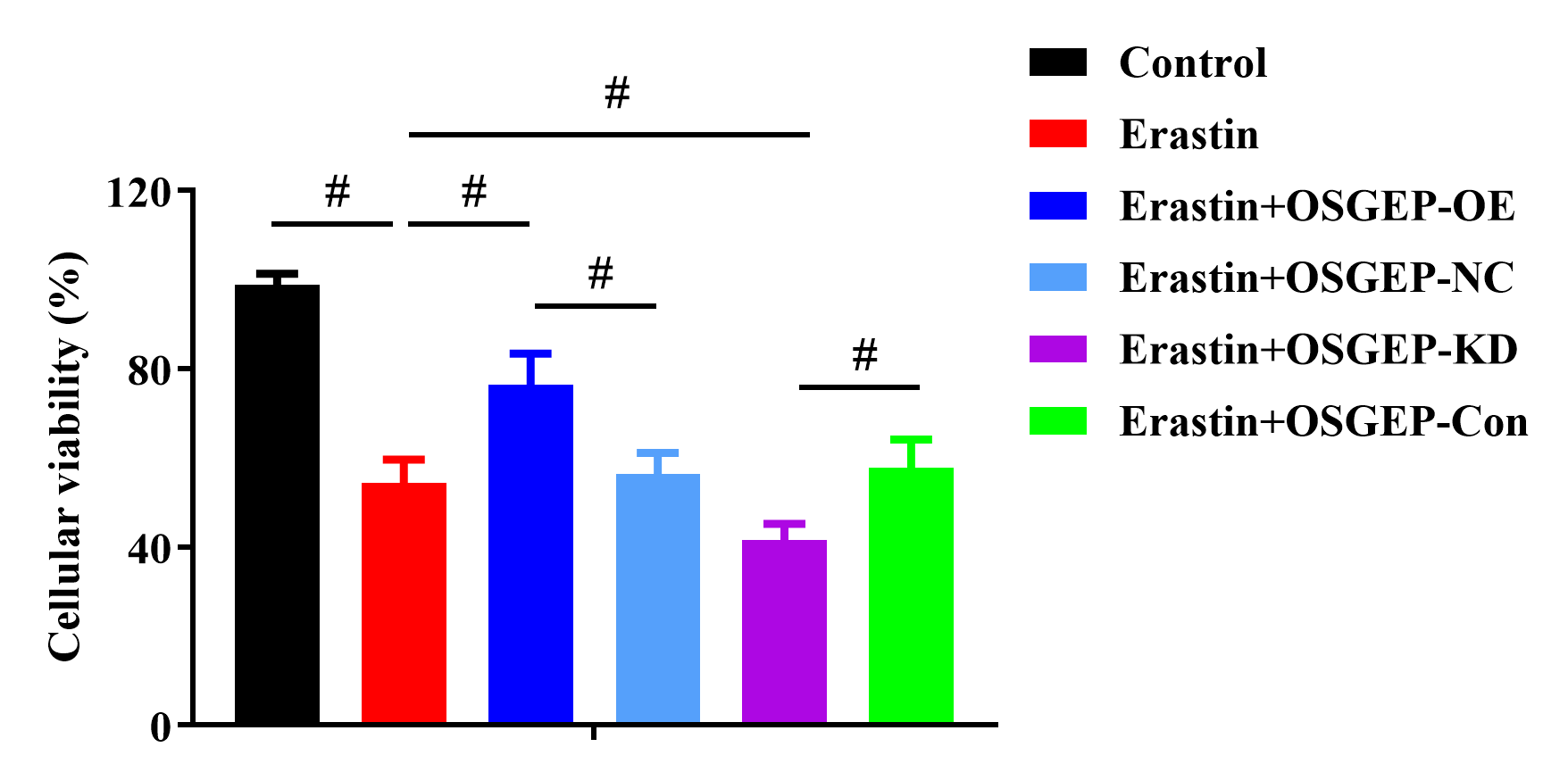
**

**S12.**

**
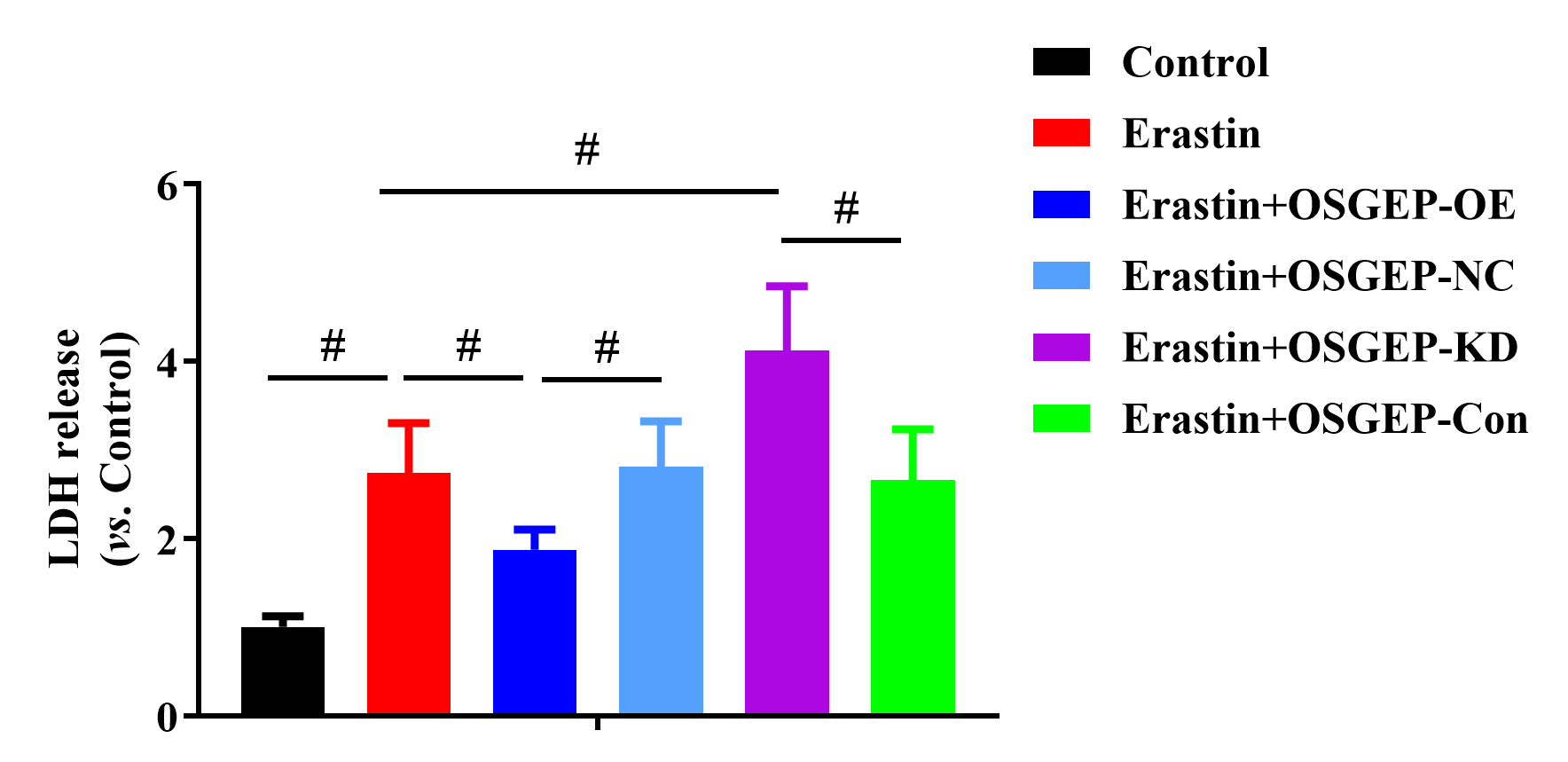
**

(**S11**) and (**S12**) Erastin markedly suppressed the cell viability and promoted LDH leakage in hepG2 cells, while these effects were abolished by OSGEP overexpression and exacerbated by OSGEP consumption.

**S13.**


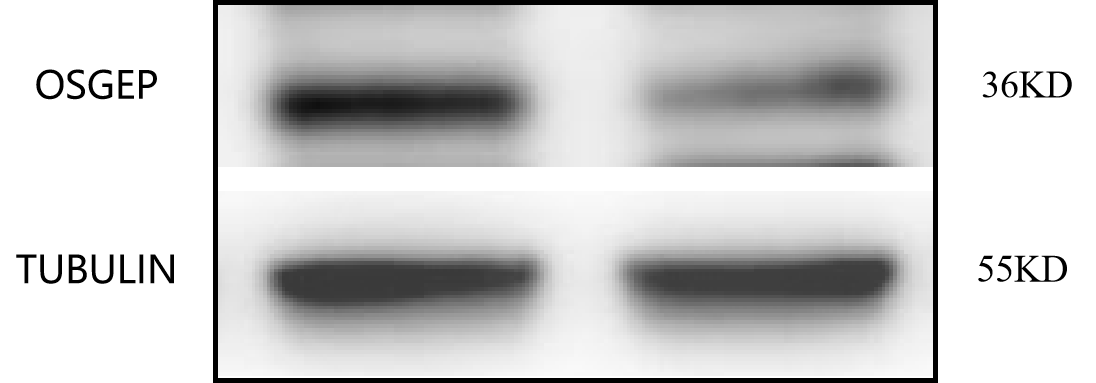


**
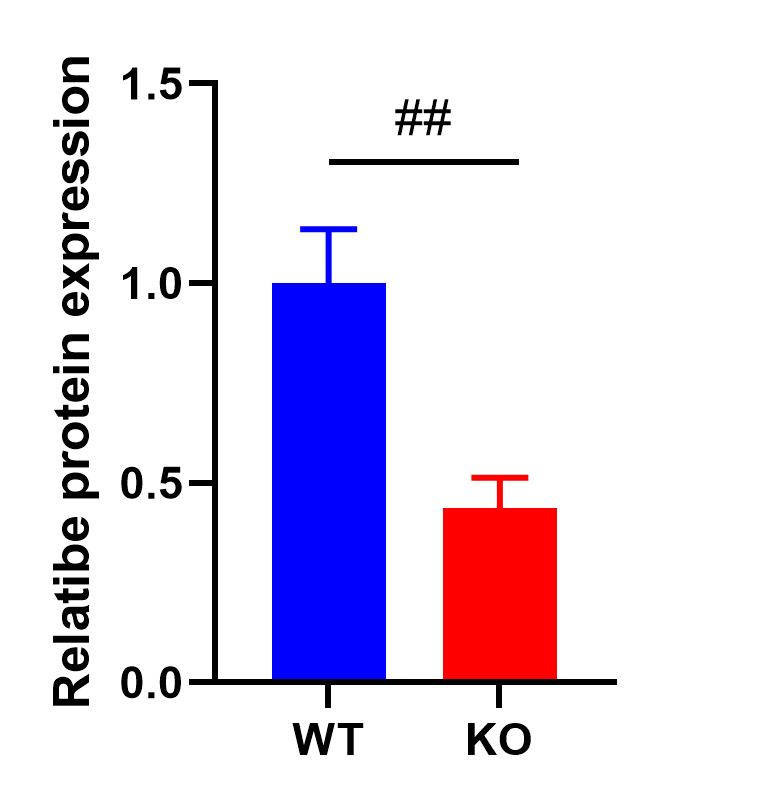
**

**(S13) the effective knockout of OSGEP in the OSGEP-KO mice**

**S14.**


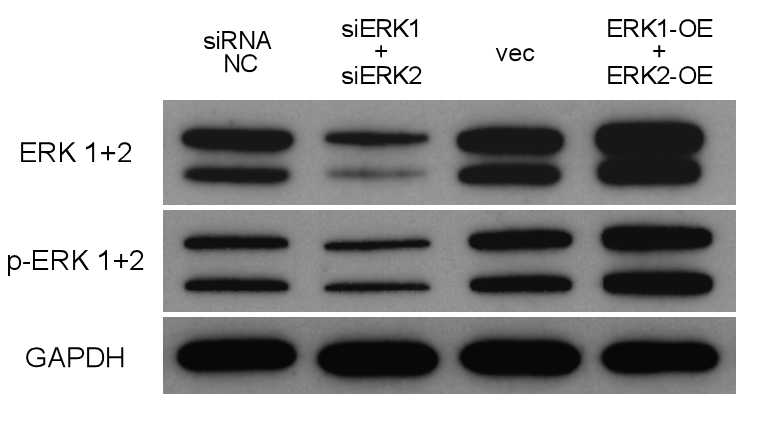


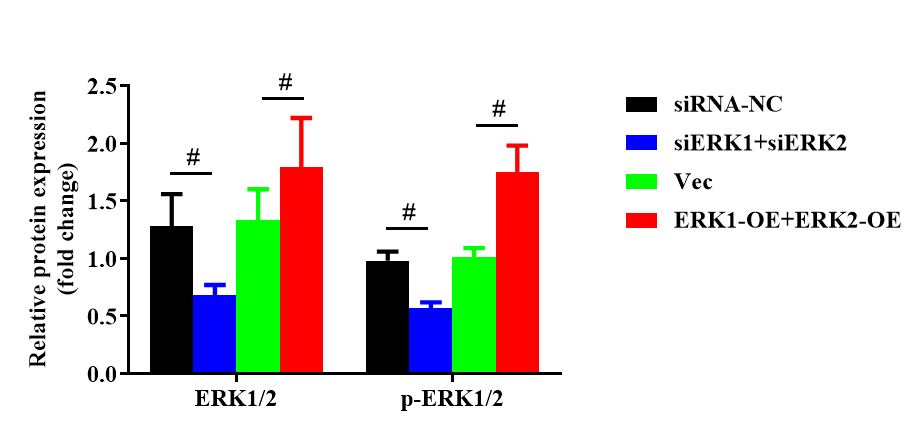


(**S14**) ERK1/2 knockdown and overexpression efficiency were detected by Western blotting. Transfection of HepG2 cells with ERK1/2 plasmid or siRNA was undertaken for 2 days, followed by exposure to OGD/R. NC: negative control for siRNA; VEC: negative control vehicle for pcDNA 3.1 vector.

**S15.**

**
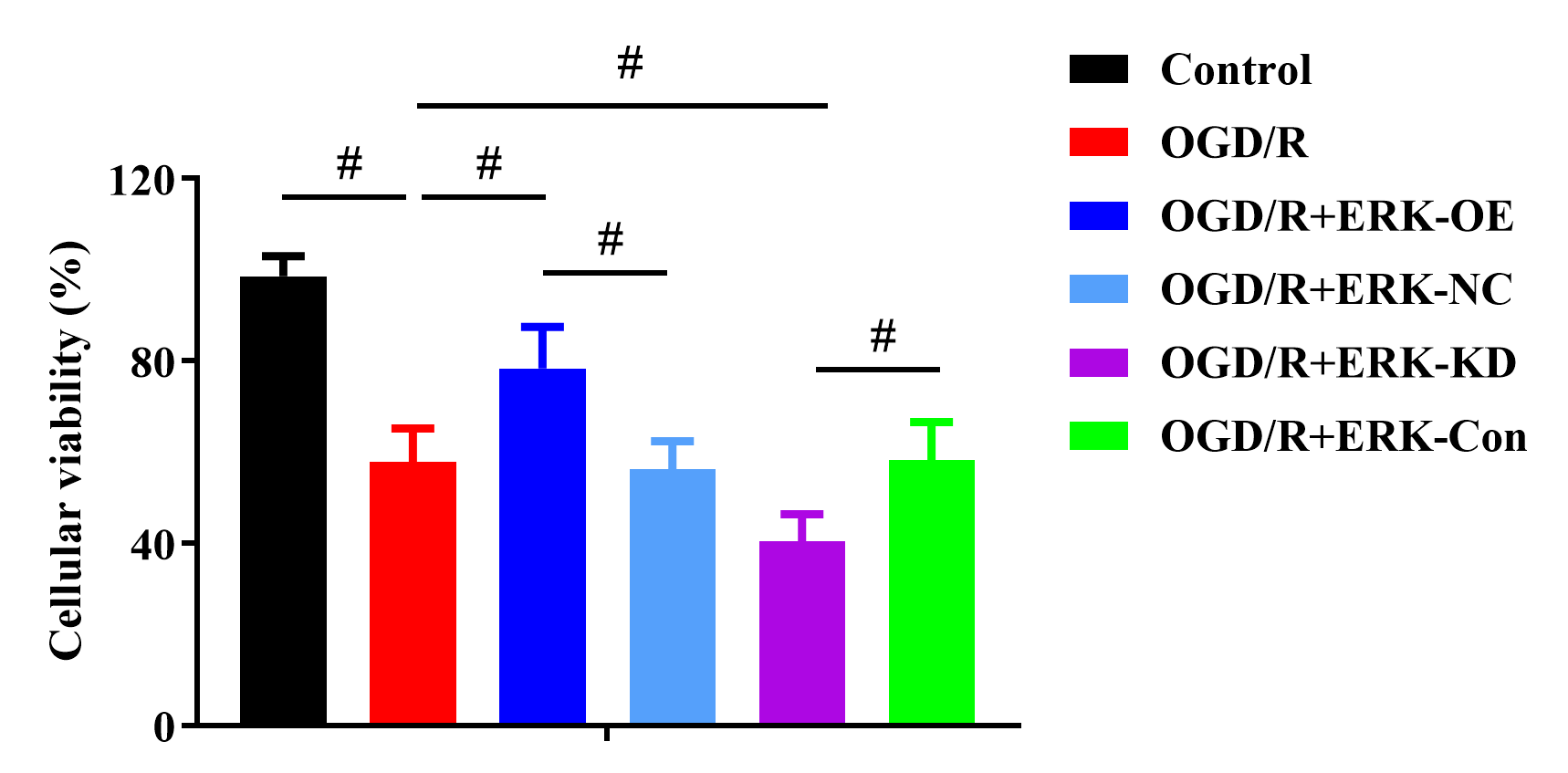
**

**S16.**

**
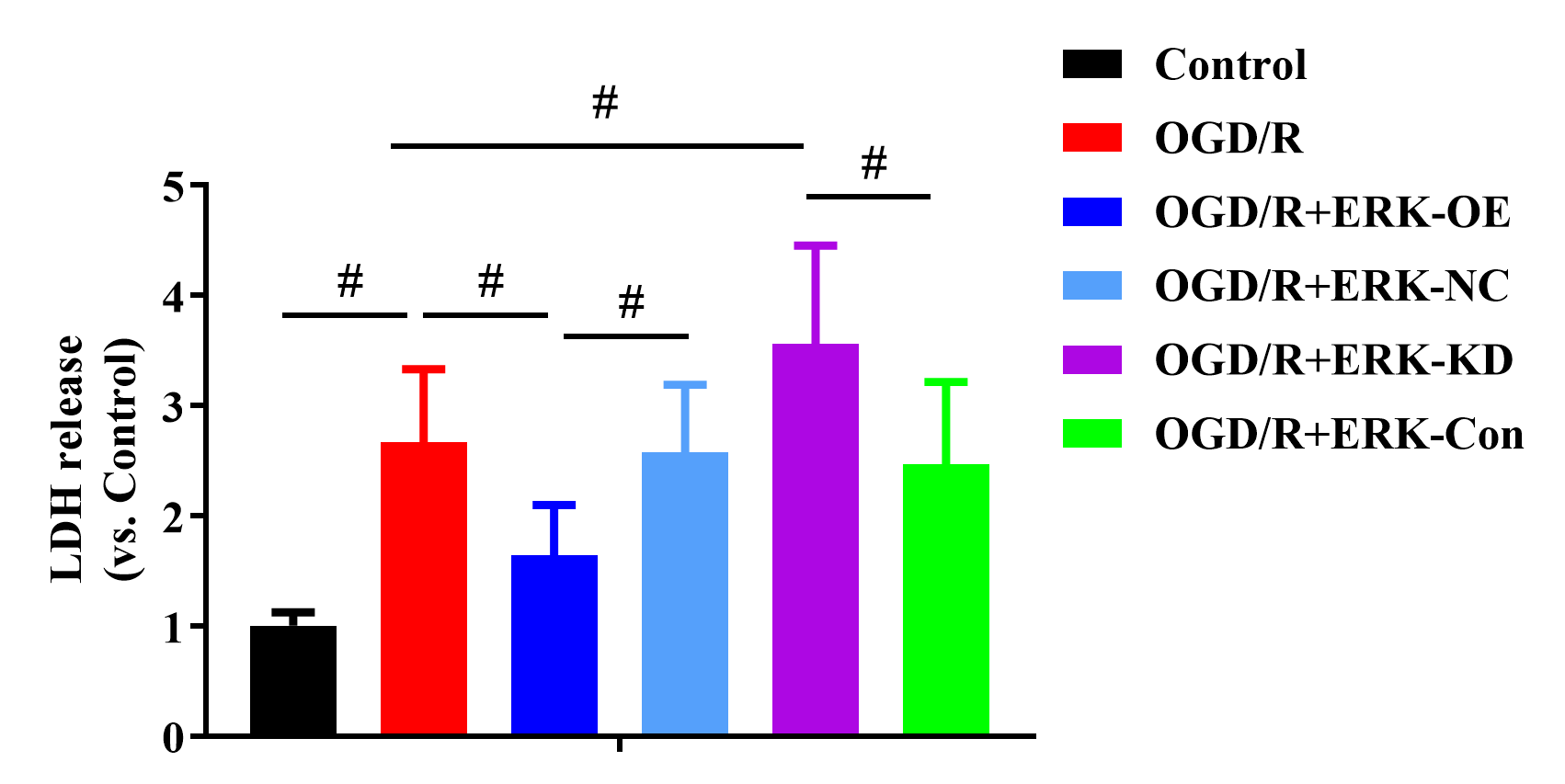
**

(**S15**) and (**S16**) The effects of ERK1/2 overexpression and knockdown on hypoxia/reoxygenation-induced cell death could be determined by CCK-8 and LDH assays.

**S17.**

**
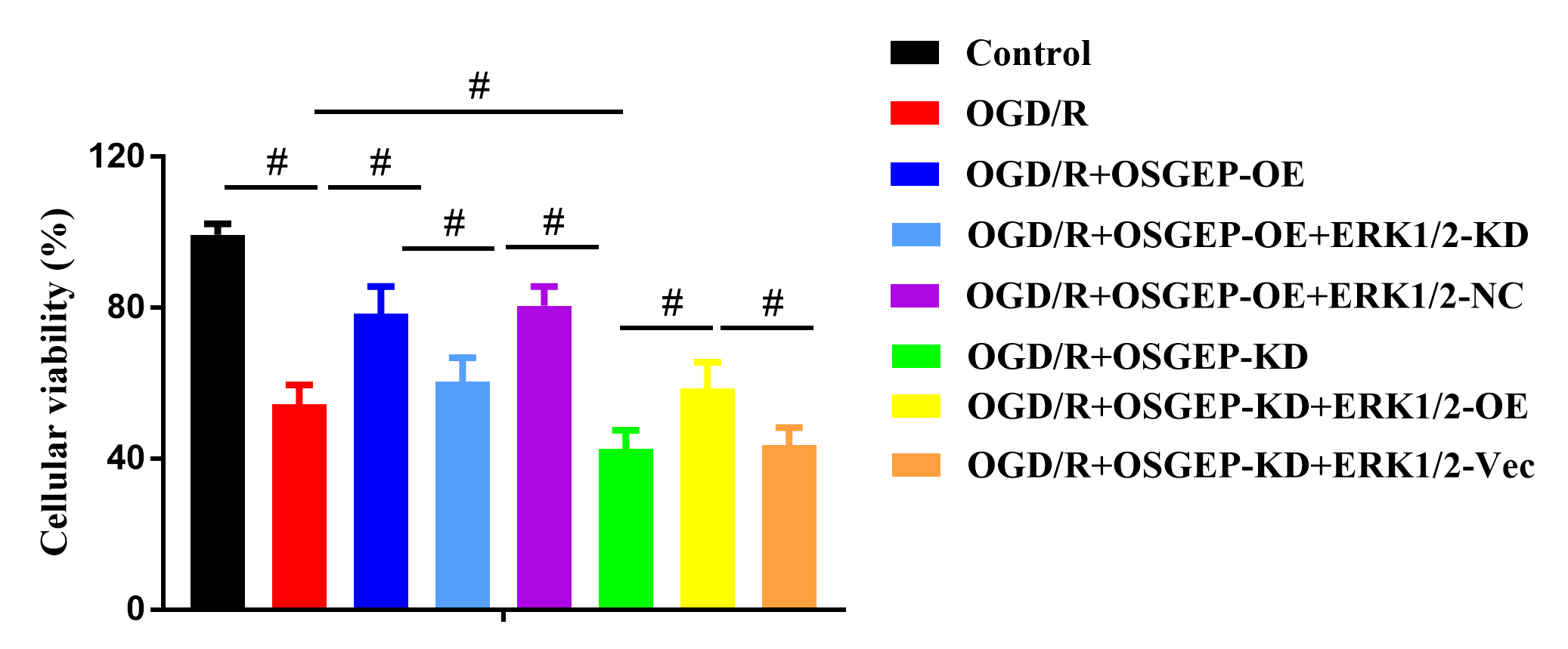
**

**S18.**

**
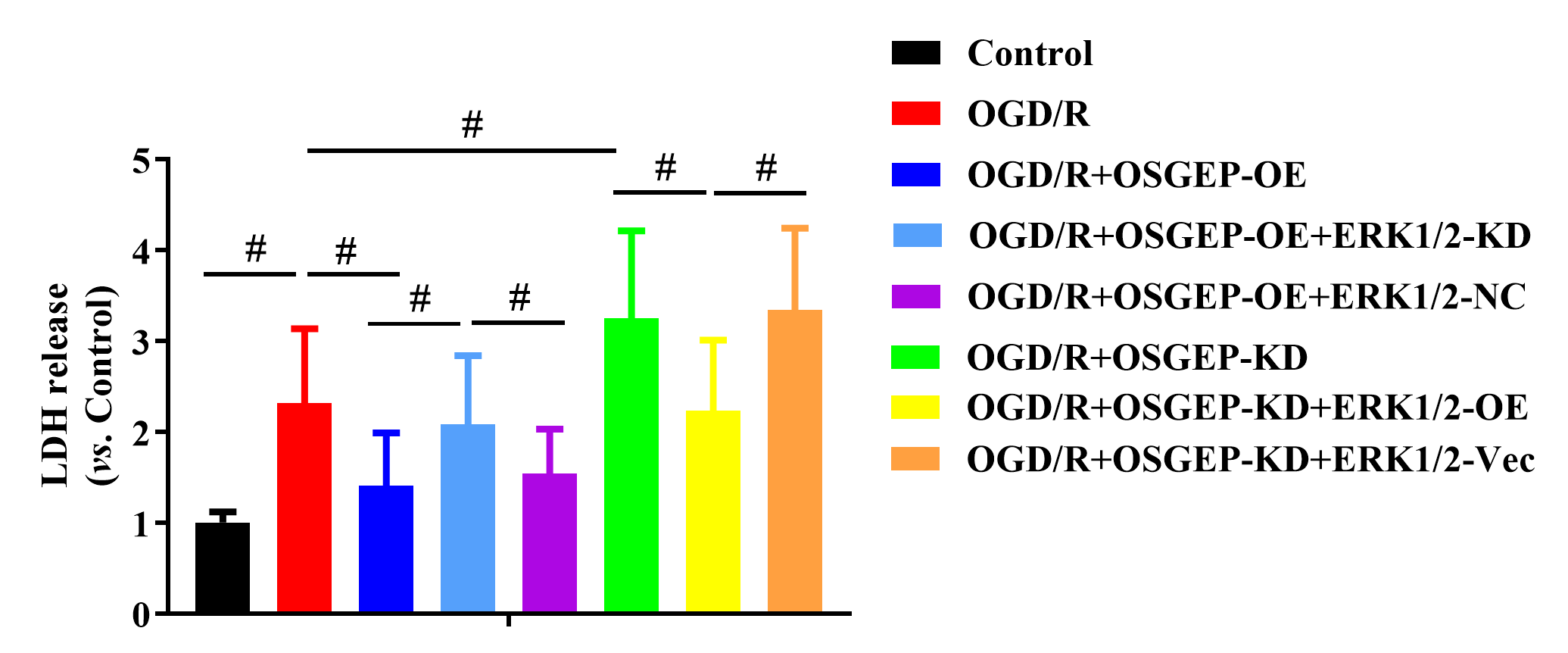
**

(**S17**) Cell viability and (**S18**) LDH release were detected.
